# Design and Validation of the Multidimensional School Social Climate Inventory for Adolescents (MSSCI-A) in Chile

**DOI:** 10.3390/bs15111588

**Published:** 2025-11-19

**Authors:** Mónica Bravo-Sanzana, Oscar Terán-Mendoza, Rafael Miranda, Xavier Oriol, Jorge Varela, Manuel Mieres-Chacaltana

**Affiliations:** 1 Núcleo Científico-Tecnológico en Ciencias Sociales y Humanidades, Universidad de La Frontera, Temuco 4780000, Chile; 2 Departamento de Psicología, Facultad de Ciencias de La Salud, Universidad Católica de Temuco, Temuco 4780000, Chile; 3 Departamento de Psicología, Universidad de Girona, 17004 Girona, Spain; 4 Facultad de Psicología, Universidad del Desarrollo, Santiago 7610658, Chile; 5 Departamento de Diversidad y Educación Intercultural, Universidad Católica de Temuco, Temuco 4780000, Chile; mieres@uct.cl

**Keywords:** school social climate, adolescents, validation, psychometrics, factor analysis, Exploratory Graph Analysis, Latin America

## Abstract

In Latin America, there is a critical need for validated instruments that capture the multidimensionality of school social climate from an ecological perspective. To fill this gap, this study developed and validated the Multidimensional School Social Climate Inventory for Adolescents (MSSCI-A). Using a non-experimental, cross-sectional design, data were collected from 8949 students across 16 Chilean regions, randomly divided for exploratory and confirmatory analyses. Content validity was ensured through expert judgment with Aiken’s V, while item variability was tested in a pilot study. Dimensionality was examined through Exploratory Factor Analysis (EFA) and Exploratory Graph Analysis (EGA), which identified 10 coherent and parsimonious dimensions. These combined expected domains such as Emotional Safety, Collaboration, and Belonging with hybrid factors that reflect how students experience interdependent aspects of school life. The Organizational Structure factor, however, did not meet minimum psychometric standards and was therefore removed. The final model was tested with Confirmatory Factor Analysis (CFA), while internal consistency and convergent validity were supported through Cronbach’s alpha, McDonald’s omega, and AVE. Findings show that the MSSCI-A demonstrates strong psychometric properties. Overall, it constitutes one of the few validated, multidimensional, and culturally grounded tools in Latin America, with applications in research, educational practice, and policymaking.

## 1. Background

Adolescence is a crucial stage in human development, where the school environment takes on particular importance. During this period, relationships with peers and significant adults outside the family are central to the formation of identity, autonomy, and a sense of belonging (Wang & Eccles, 2012). The school social climate, therefore, becomes an ecological factor that can either protect against psychosocial risks or, on the contrary, generate vulnerability (Franco et al., 2023; Podiya et al., 2025; World Health Organization, 2018). The school is thus positioned as a key environment for promoting student well-being, preventing violence, and strengthening socioemotional skills (Wang & Degol, 2016; Yamaguchi et al., 2024).

A growing body of recent empirical studies shows that school social climate in adolescence is significantly associated with mental health and well-being (Aldridge & McChesney, 2018; Franco et al., 2023; Lucas-Molina et al., 2025; Urke et al., 2023; Wong et al., 2021; Yamaguchi et al., 2024). For instance, bullying victimization has been identified as one of the most influential and negative indicators on students’ subjective well-being (Oriol et al., 2025). Similarly, network analyses have revealed that cyberbullying and exclusion have negative long-term relationships with adolescent self-esteem and well-being (Vieta-Piferrer et al., 2024).

Despite the vast evidence, measuring school social climate in adolescence faces significant methodological challenges, primarily due to its multidimensional nature. Additionally, there is a need for instruments that are not only rigorously validated but also comparable and sensitive to diverse sociocultural contexts, such as those found in Latin America. Consequently, the primary objective of this research is to develop and validate a multidimensional measure of school social climate developed explicitly for adolescents in the Chilean context.

### 1.1. Bronfenbrenner’s Ecological Approach as a Foundational Framework for the School Social Climate

The analysis of school social climate is framed within a theoretical tradition that recognizes the contextual and multidimensional nature of educational experiences. Among the most influential theories is Bronfenbrenner’s (1987, 1989, 1992) ecological model, which posits that human development is determined by reciprocal interactions between the individual and the multiple systems in which they are embedded, ranging from the immediate microsystem (such as family, classroom, or school) to the broader cultural macrosystem. This approach is particularly relevant for understanding school social climate, as it allows for an analysis of how the dynamics among school actors, institutional policies, and organizational characteristics interact to shape perceptions of safety, belonging, social support, and learning opportunities (M. Bravo-Sanzana et al., 2019a, 2019b; Cohen et al., 2009; Eccles & Roeser, 2011; Trianes et al., 2006; Wang & Degol, 2016).

Bronfenbrenner (1987, 1989, 1992) argues that educational environments do not operate in isolation but are interrelated and mutually influential, creating conditions that either promote or inhibit student well-being. In this sense, school climate can be understood as an emergent manifestation of the school microsystem, modulated by influences from the exosystem (e.g., public policies) and the macrosystem (shared cultural values). This relational and contextual perspective has been adopted by various recent studies on school climate (M. Bravo-Sanzana et al., 2020a, 2020b; Grazia & Molinari, 2021; Rudasill et al., 2018; Wang & Degol, 2016), which agree on the importance of analyzing the construct as an ecologically situated and socially constructed phenomenon.

From this ecological perspective, the need arises for school social climate measurement instruments that are consistent with the construct’s complexity and multicausality. Since school social climate emerges from the dynamic interaction among actors, organizational structures, and macrosocial contexts, its evaluation must be sensitive to this plurality, incorporating multiple levels of analysis (classroom, school, system) and diverse informants (students, teachers, educational assistants, administrators, families). Furthermore, the measures must be supported by rigorous psychometric evidence that guarantees their construct validity, internal reliability, and cultural relevance. Only through instruments that capture the relational, situated, and contextual nature of school social climate (M. V. Bravo-Sanzana et al., 2023; Grazia & Molinari, 2021; Wang & Degol, 2016) will it be possible to use this information to guide educational policies, school improvement processes, and strategies for promoting comprehensive well-being, in line with the principles of Bronfenbrenner’s ecological model.

### 1.2. School Social Climate: Conceptualization and Multidimensional Structure

School social climate (SSC) is a broad construct defined as the shared perceptions of an educational community regarding the social, emotional, academic, and institutional aspects of the school environment (Cohen et al., 2009; Grazia & Molinari, 2021; Wang & Degol, 2016). While no single, universally agreed-upon definition exists, there is consensus that it is a multidimensional construct. Its complexity necessitates a comprehensive approach that incorporates both interpersonal interactions and the norms, values, beliefs, and organizational structures present in a school (M. V. Bravo-Sanzana et al., 2023; Rudasill et al., 2018).

Wang and Degol (2016) propose organizing SSC into four fundamental domains: (1) academic environment, (2) community environment, (3) safety environment, and (4) institutional environment. This classification enables the analytical systematization of the various dimensions that comprise school climate, facilitating its measurement and comparative analysis across different educational settings. Table 1 provides a detailed description of these four general domains, along with their respective dimensions.

This organization is structured into four domains and 13 dimensions (12 for students), which have been empirically validated and are one of the most influential and widely replicated proposals internationally (Grazia & Molinari, 2021; Wang & Degol, 2016).

### 1.3. Measuring the Social Climate in Latin American Schools: Conceptual and Psychometric Challenges

The evaluation of school social climate has evolved from unidimensional, student-centered approaches to more complex models that consider its multidimensionality, subjective nature, and relational character. The systematic review by M. V. Bravo-Sanzana et al. (2023), based on the PRISMA methodology and COSMIN criteria, provides evidence that the instruments used in Latin America have significant limitations. In their analysis of 27 studies, they identified that most instruments include the community (100%) and safety (93%) domains, as well as the academic domain (85%). However, only 30% incorporate dimensions of the institutional environment, despite its relevance for school management. Additionally, many scales lack rigorous evaluations of structural validity, internal consistency, and cross-cultural validity. Furthermore, content validity was found to be inadequate in most of them due to a lack of information (e.g., expert judges, evaluation criteria, and translation processes of the scales).

Wang and Degol (2016) had previously warned about the need to move toward validated, comparable, and culturally sensitive instruments. They agree with M. V. Bravo-Sanzana et al. (2023) on the need to adopt a multidimensional approach to capture the complexity of the phenomenon. This type of approach fosters a more comprehensive understanding and allows for the establishment of relationships between SSC and other relevant outcomes such as adolescent well-being (Oriol et al., 2025; Vieta-Piferrer et al., 2024), mental health (Aldridge & McChesney, 2018; Franco et al., 2023; Lucas-Molina et al., 2025; Urke et al., 2023; Wong et al., 2021; Yamaguchi et al., 2024), and academic performance (M. Bravo-Sanzana et al., 2021; X. Chen & Liao, 2025; Chzhen & Leesch, 2023; Thapa et al., 2013).

The challenges of measurement are not only technical but also epistemological, as they require a clear definition of the constructs involved, the establishment of robust conceptual frameworks, and assurance that instruments are culturally relevant. In Latin America, the concept of school coexistence (*convivencia escolar*, in Spanish) is frequently used as a synonym for school social climate, integrating a perspective of rights, inclusion, and violence prevention (Del Rey et al., 2009). This cultural specificity requires that instruments not only translate international scales but also incorporate dimensions specific to the local context.

### 1.4. The Present Study

In this scenario, the need is evident for validated measurement instruments that, from an ecological perspective, can capture the multidimensionality of school social climate, its different levels of analysis, and the interaction among its components. The instrument must be sensitive to the growing complexity of adolescents’ perceptions, their critical capacity, and their voice, while also demonstrating evidence of structural validity and reliability for this age group.

Additionally, in line with Bronfenbrenner’s (1987, 1989, 1992) ecological approach, measuring school climate in adolescence should integrate variables related to student agency, participation in decision-making, and perceptions of school justice. These elements, which are particularly relevant in this developmental stage, require instruments that reflect this systemic complexity.

Consequently, the general objective of this study was to develop and validate a multidimensional scale of school social climate for adolescents in Chile. The specific objectives proposed were: (1) To develop pertinent and coherent indicators with the multidimensional model of school social climate; (2) To determine the content validity through expert judge evaluation; (3) To establish the factorial structure of the scale as evidence of construct validity; (4) To estimate the instrument’s reliability through measures of internal consistency; (5) To determine the scale’s invariance by gender.

## 2. Materials and Methods

### 2.1. Participants and Design

This study used a non-experimental, cross-sectional design with an instrumental scope, as its central objective was to develop and validate a multidimensional measure of school social climate.

The total sample consisted of 8949 students from 16 regions of Chile, selected using a non-probabilistic cluster sampling method. Of these, 311 participants took part in the pilot study, while the remaining sample (*n* = 8638) was randomly divided into two subsamples: 4319 students for the exploratory factor analysis (EFA) and 4319 for the confirmatory factor analysis (CFA).

### 2.2. Instruments

#### The Multidimensional School Social Climate Inventory for Adolescents

For this study, the Multidimensional Scale of School Social Climate for students (MSSCI-S) was developed. The process began with a systematic literature review (M. V. Bravo-Sanzana et al., 2023) to identify instruments that measure the dimensions of school social climate proposed by Wang and Degol (2016) and validated by Grazia and Molinari (2021).

From this review, relevant indicators for the 13 theoretical dimensions were extracted, and culturally relevant versions of the items were developed. This was accomplished by a collaborative committee comprising two experts in psychological measurement and evaluation, as well as two researchers from the core team. The initial item bank consisted of 109 items. The response format is mixed and adjusted to the nature of each item. Therefore, some of them are answered on a frequency scale (from 0 = never to 4 = always), others indicate the level of agreement (from 0 = totally disagree to 4 = totally agree), and a smaller proportion are answered dichotomously (0 = No, 1 = Yes). The validity and reliability of the scale will be described in the results section.

### 2.3. Procedure

First, the preliminary item bank was shared with a committee of five expert judges (two psychologists, one social scientist, one anthropologist, and one school counselor). They evaluated the preliminary version of the items using the criteria of clarity, coherence, relevance, and sufficiency, as proposed by Escobar-Pérez and Cuervo-Martínez (2008) for content validity analysis. The evaluation used a scale from 0 (Insufficient) to 3 (Sufficient), and experts were able to provide recommendations in a comments box. Based on this feedback and statistical analysis, adjustments were made to the scale’s content (Ventura-León, 2022).

Subsequently, a pilot test was conducted to gather usability feedback on the instrument, assess the comprehension of the questions and response scales, and identify any potentially problematic questions (Muñiz & Fonseca-Pedrero, 2019). Following this process, adjustments were made as needed before proceeding with the formal administration of the questionnaire.

Initial contact with the schools was established through a national outreach campaign via email and phone, from March to October 2024. The purpose was to invite school management and school coexistence teams to a virtual meeting to present the project. Official project materials were utilized to outline the objectives, scope, tentative timeline, anticipated benefits, and ethical safeguards. During this meeting, the approval document from the Scientific Ethics Committee (CEC) (code: 027/22) was provided, operational questions were addressed, and each school designated a contact person for ongoing coordination.

Schools that chose to participate signed an institutional Letter of Commitment, signed by the principal (and the sponsor, when applicable). This letter explicitly authorized the implementation of the survey, specified the necessary resources (including space and time), and outlined data protection agreements. In accordance with current regulations, the required informed consent forms (institutional, parental, and student assent) were managed using formats provided by the research team.

Before administering the survey, the research team provided technical guidance to the designated school staff (inspectors, administering teachers, or IT coordinators) via a synchronous session and an implementation manual. The guidance covered the following points: (a) multi-informant inclusion criteria (students regularly enrolled in target courses and school adults by role); (b) the in-room protocol (seating arrangement, timing, instruction reading, and question handling); (c) the support route for incidents; and (d) the procedure for ensuring the anonymity and voluntary nature of responses.

### 2.4. Data Analysis

For the content validity analysis, item averages were calculated for each dimension, with an item meeting the criterion if its average was greater than 2. Inter-judge agreement was calculated using Aiken’s V, with a value greater than 0.80 considered acceptable (Ventura-León, 2022). For the pilot test, the variability of the items in the response scale was evaluated to ensure that there were no items without variability or response options left empty (Muñiz & Fonseca-Pedrero, 2019).

Regarding the exploratory analysis, two approaches were used. The first, based on classical test theory, consisted of an Exploratory Factor Analysis (EFA) with a mixed correlation matrix (polychoric/tetrachoric), given the ordinal and dichotomous nature of the response scales (Ferrando & Lorenzo-Seva, 2014). Basic assumptions for factor analysis were verified, including the Kaiser–Meyer–Olkin (KMO) criterion, with a value above 0.80 considered acceptable, and a statistically significant result from Bartlett’s test of sphericity. To determine the number of factors to retain, a parallel analysis was conducted, with only those factors exceeding 95% of the simulated structures considered admissible. Estimation was performed using a weighted least squares method, and the matrix rotation was performed using direct Oblimin, considering that the factors are conceptually correlated (Lloret-Segura et al., 2014).

Additionally, a psychometric network analysis approach was implemented to explore and validate the scale’s dimensionality. Specifically, an Exploratory Graph Analysis (EGA) was applied (Christensen & Golino, 2021; Golino & Epskamp, 2017). This technique identifies the number of latent dimensions based on the network structure of regularized partial correlations. This approach uses the Graphical Least Absolute Shrinkage and Selection Operator (Glasso) estimation model to control network complexity and obtain a more stable representation of the relationships between items. Subsequently, the identification of item communities, which correspond to potential factors, was performed using the Louvain algorithm, a widely used method for detecting groups in complex networks. In both cases, the retention of items depended on three criteria: (1) a factor loading greater than 0.300; (2) no cross-loading (an item with loadings above 0.300 on more than one factor); and (3) the item being theoretically congruent with the factor to which it was assigned.

Since the indicators obtained from the EFA and EGA are not directly comparable, as they are based on different assumptions and metrics, the decision of which model to retain was based on conceptual and interpretability criteria rather than on a direct comparison of statistical indicators. Consequently, the most parsimonious and conceptually congruent model was chosen, which had to offer an adequate representation of the data with a reduced number of dimensions and maintain a close correspondence with the theoretical model proposed by Wang and Degol (2016). This choice ensures theoretical coherence and enhances the interpretability of the results within the existing literature framework.

Subsequently, the selected factorial model was tested with a confirmatory factor analysis (CFA) based on structural equation models (SEM). Model fit was evaluated using commonly accepted goodness-of-fit indices, according to the criteria proposed in the literature (Hu & Bentler, 1999; Kline, 2016). The Weighted Least Squares Mean and Variance adjusted (WLSMV) estimator was used, as it is appropriate for ordinal variables. A model was considered to have an adequate fit when the Comparative Fit Index (CFI) and the Tucker–Lewis Index (TLI) were equal to or greater than 0.95, the Root Mean Square Error of Approximation (RMSEA) was ≤0.06 along with its 90% confidence interval, and the Standardized Root Mean Square Residual (SRMR) was ≤0.08. Standardized factor loadings were evaluated with a minimum threshold of ≥0.400, a criterion that ensures substantive associations between the items and their underlying factors, guaranteeing that each indicator contributes in a theoretically relevant way to the measured construct.

The internal consistency of the dimensions was evaluated using Cronbach’s alpha (α) and McDonald’s omega (ω), both reliability indicators that reflect the degree to which a scale’s items relate to each other and to the underlying construct. In both cases, values equal to or greater than 0.70 are considered acceptable, while figures above 0.80 suggest high reliability (Kalkbrenner, 2021). Furthermore, the Average Variance Extracted (AVE) was used to examine the convergent validity of the dimensions. An AVE value of ≥0.50 indicates that the construct explains at least 50% of the variance of its items, which provides adequate evidence of convergence (Hair et al., 2019).

To examine the equivalence of the measurement model across groups, a multigroup confirmatory factor analysis (MCFA) was conducted following the recommendations of Putnick and Bornstein (2016). Specifically, metric and scalar invariance were evaluated. Metric invariance was tested by constraining factor loadings to equality across groups, ensuring that the latent constructs have the same meaning and scale of measurement. Scalar invariance was tested by additionally constraining item intercepts to equality, which allows for meaningful comparisons of latent means across groups.

Invariance was further evaluated by comparing nested models, with ΔCFI ≤ 0.01 and ΔRMSEA ≤ 0.015 as thresholds, suggesting that additional constraints did not result in significant deterioration of fit (F. F. Chen, 2007).

The data were collected using the LimeSurvey platform. All statistical analyses were performed using RStudio v 2025.05.0+496 (Posit Team, 2025). The following packages were used for the analyses: the *psych* package v2.5.3 for descriptive statistics and the EFA; the *EGAnet* package v2.3.0 for the EGA; the *lavaan* package v0.6.19 for CFA and invariance testing; the *semTools* package v0.5.7 to compute reliability coefficients and AVE.

## 3. Results

### 3.1. Content Validity

The instrument’s refinement process was based on a detailed evaluation by expert judges. This process involved a series of adjustments to its dimensions to improve the validity and relevance of the items. For a more comprehensive overview of these changes, please refer to Supplementary Material S1.

In the Emotional Security dimension, the objective was to improve the parsimony of items. The content of item 4 was disaggregated to differentiate between emotions and thoughts, as well as to distinguish between teachers and management staff. Item 5 was made more specific to inquire about peers, while the remaining items were left for teachers and management teams. For the Discipline and Order dimension, the first item was disaggregated into two. The second item was eliminated due to the impracticality of a student evaluating another’s knowledge. Item 3 was divided into two to address separately the perception of the utility of rules and their compliance. Redundant words were removed from items 4 and 6, and an item about the application of sanctions by management staff was added.

For Physical Security, adjustments were made to specific items, separating contents such as bullying and cyberbullying. An item that evaluates situations of threats and insults between adults was incorporated, and two items with low scores from the expert judges were eliminated. The Collaboration dimension underwent terminological adjustments in items. Expressions like “my family” were modified to “my guardian (*apoderado/a*, in Spanish),” and the contents were divided to differentiate between academic help and help with personal problems. Items about collaboration in cleaning tasks were replaced by those addressing participation in institutional management (e.g., student council). The item on the quality of general relationships was divided into three.

In Sense of Belonging, an item was added, as suggested by the judges, to explore the level of identification students have with the educational project. For Respect for Diversity, the number of items was maintained, with specific semantic adjustments. In Leadership, terms were modified, and an item was eliminated due to conceptual redundancy. In the Teaching dimension, the term “reprimand” was replaced with “call attention to.”

In Physical Environment, the item that asked whether the school was a generally comfortable place was eliminated, as it measured a general domain and could overestimate variance. Finally, in Organizational Structure, minor wording adjustments were made without adding or eliminating items. Dichotomous questions about the availability of resources were removed to focus the evaluation on the quality and sufficiency of these. Additionally, an item was added that inquires about the availability of a social worker. The number of items in the preliminary version and the adjusted version after review by judges can be seen in Table 2.

Finally, the 118-item version was applied to a pilot sample to ensure there were no comprehension issues. Feedback from participants indicated that there were no usability problems with either the scale or the platform used.

### 3.2. Structural Validity

#### 3.2.1. Exploratory Factor Analysis

The univariate descriptive statistics for each item are found in Supplementary Material S2. To determine the optimal number of factors to retain in the Exploratory Factor Analysis (EFA), the parallel analysis method was used (Horn, 1965), one of the most reliable techniques for this purpose, with methodological optimizations developed in recent years (Lorenzo-Seva et al., 2011; Timmerman & Lorenzo-Seva, 2011). The results of the parallel analysis suggested a 22-factor solution. However, retaining such a high number of factors was deemed unfeasible from both psychometric and theoretical perspectives.

First, a model with 22 factors would lack parsimony and its interpretation would be highly complex and, in practice, unviable (Preacher & MacCallum, 2003). Each factor would explain only a very small portion of the total variance, which would hinder the assignment of a clear theoretical meaning and the generalizability of the findings (Brown, 2015). More importantly, a 22-factor solution categorically contradicts the conceptual framework of this study, which postulates that school social climate is a multidimensional construct structured into a limited number of previously validated domains and dimensions.

Based on these considerations, the result of the parallel analysis was interpreted as an overfitting of the sample variance. Consequently, given the need to explore the structure of a complex construct with a more holistic and less restrictive perspective than traditional EFA (Golino et al., 2022), Exploratory Graph Analysis (EGA) was tested as an alternative for exploring the factor structure of the scale while controlling for variance.

#### 3.2.2. Exploratory Graph Analysis

EGA identifies latent structure based on the clustering of highly correlated items, often resulting in a clearer and more replicable representation of the construct (Golino & Epskamp, 2017). EGA was fitted using the GLASSO model, which, unlike EFA, applies a regularization technique to obtain a more stable partial correlation matrix, producing a more faithful and less overfitted representation. This approach identified a total of 11 communities (or latent dimensions), a solution that is significantly more parsimonious and consistent with the conceptual framework of the construct. The loading magnitude of each item on its assigned community is listed in Supplementary Material S3.

Conceptually, the communities identified by EGA largely correspond consistently with the dimensions of the Wang and Degol (2016) model, providing strong evidence of construct validity. However, in some communities, EGA revealed item groupings that diverged from the original model.

Specifically, some communities aligned precisely with the theoretical model: Emotional Safety (Community 1), Collaboration (Community 4), Interpersonal Relationships (Community 5), Sense of Belonging (Community 6), and Resource Availability (Community 11) emerged as clearly defined factors, with all their theoretically assigned items grouped in a single community. Similarly, the Teaching and Learning dimension (Community 8) and items related to Organizational Structure Management (Community 10) appeared practically pure.

Nevertheless, EGA also identified the integration of items from different dimensions within other communities, suggesting that, for the studied population, these perceptions are intertwined in practice: Discipline, Order and Physical Safety (Community 2); Physical Safety and Respect for Diversity (Community 3); Respect for Diversity and Leadership (Community 7); Physical Environment and Objective Organizational Structure (Community 9). Finally, 17 items were removed because they did not reach the loading cutoff of 0.300 in any of the communities.

#### 3.2.3. Confirmatory Factor Analysis

To assess the fit of the structure identified in the EGA, a CFA was conducted, including the 11 factors under an initial first-order model in which the latent variables were freely correlated. The model was estimated using the weighted least squares with mean and variance adjustment (WLSMV) method, with freely estimated standardized loadings and robust standard errors. The results indicated a good model fit to the data, with indices exceeding conventional thresholds commonly accepted for large samples and multidimensional scales: CFI = 0.973, TLI = 0.972, RMSEA = 0.042 (90% CI [0.041, 0.042], *p* < 0.001), and SRMR = 0.049. A robust RMSEA of 0.043 was also observed, with consistent values of robust CFI and TLI (CFI = 0.971, TLI = 0.970), confirming the stability of the model fit under non-normality correction.

All factor loadings were statistically significant (*p* < 0.001), with standardized values ranging from 0.403 to 0.883. Overall, items showed high loadings on their corresponding factors, suggesting adequate empirical convergence. However, the factor associated with organizational structure exhibited moderate loadings (λ = 0.454–0.580), which may reflect students’ lower perceived clarity regarding these aspects or greater heterogeneity in their measurement. The covariance matrix between factors is presented in Table 3.

#### 3.2.4. Reliability and Variance Extracted

The results of reliability and convergent validity analyses indicated adequate levels of internal consistency across most dimensions. In particular, Cronbach’s alpha and McDonald’s omega coefficients exceeded 0.85 in nearly all factors, reaching excellent values in dimensions such as Sense of Belonging (α = 0.922; ω = 0.920) and Respect for Diversity + Leadership (α = 0.946; ω = 0.945), demonstrating high item homogeneity. However, the Organizational Structure factor showed considerably low values (α = 0.618; ω = 0.621), indicating insufficient internal consistency (see Table 3).

Regarding convergent validity, the analysis of the Average Variance Extracted (AVE) indicated that only a few factors (F5, F6, F7, and F8) exceeded the recommended threshold of 0.50, suggesting that the latent construct explains more than half of the variance observed in the items. In contrast, several dimensions (F1, F2, F3, F4, F9, and F11) yielded values slightly below the cutoff, though still acceptable under conditions of high reliability and expected when more than six latent factors are present (Hair et al., 2019). Given the substantially low reliability indices (α = 0.618; ω = 0.621) and the insufficient convergent validity (AVE = 0.299), the Organizational Structure factor (F10) with four items does not meet the minimum psychometric standards. Therefore, the decision was made to exclude this factor from subsequent analyses, as its inclusion could compromise the overall validity and reliability of the measurement model (see Table 4). The final version of the instrument consisted of 97 items across 10 dimensions.

### 3.3. Measurement Invariance

Table 5 presents the results of the invariance models with progressively increasing constraints. The changes in fit indices (ΔCFI ≤ 0.01; ΔRMSEA ≤ 0.015) remained below the recommended thresholds, indicating that the model achieved scalar invariance, also known as strong invariance. This result implies that both factor loadings and item intercepts can be considered equivalent across groups, thereby allowing for the comparison of latent means between men and women.

## 4. Discussion

The present study aimed to develop and validate a multidimensional instrument to assess school social climate among Chilean adolescents, grounded in Bronfenbrenner’s (1987, 1989, 1992) ecological approach and a robust theoretical structure proposed by Wang and Degol (2016). From this perspective, the MSSCI-A conceptualizes school climate as the result of dynamic interactions among individuals, relationships, and institutional systems, consistent with the ecological framework outlined earlier. The findings support the validity and reliability of the MSSCI-A, a measure designed to capture the complexity of the construct from a culturally relevant perspective.

A distinctive feature of this study was the methodological rigor applied in the item development and content validation process, which strengthens the instrument’s validity from its early stages. The item construction was based on a systematic literature review and on widely accepted theoretical frameworks, with cultural adaptations specifically tailored to the Chilean context. Subsequently, the preliminary version was evaluated by an interdisciplinary panel of expert judges, who assessed each item’s clarity, coherence, relevance, and sufficiency. This procedure aligns with the recommendations of the COSMIN framework for developing measurement instruments, which emphasizes the importance of ensuring content validity through structured and transparent procedures. The adjustments derived from this expert review enabled the refinement of the instrument’s dimensions, enhanced the semantic precision of the items, and ensured conceptual coverage across all domains.

From the perspective of the instrument’s internal structure, the factor solution obtained through EGA was more parsimonious and theoretically coherent with the model proposed by Wang and Degol (2016) and aligned with Bronfenbrenner’s ecological conception of nested systems, where multiple contextual levels converge to shape students’ experiences, in comparison to the 22-factor solution suggested by the EFA parallel analysis. EGA identified 10 communities or latent dimensions, several of which aligned precisely with the theoretical dimensions, such as Emotional Safety, Collaboration, Interpersonal Relationships, Sense of Belonging, and Resource Availability. These results provide empirical support for the structural validity of the instrument, suggesting that students’ perceptions tend to organize coherently around the normative components of school climate.

At the same time, EGA revealed the presence of hybrid communities, composed of items associated initially with different dimensions. For example, clusters combining Discipline and Physical Safety, or Respect for Diversity with Leadership, were observed. These configurations can be interpreted as a manifestation of how students integrate multiple school experiences into dimensions perceived as interdependent. From our perspective, this also reflects the concept of school coexistence management, since the aforementioned hybrid communities are composed of elements related to actions implemented within the school to promote order and ensure students’ physical integrity. Likewise, in the case of items that converge on respect for diversity and leadership, these are specifically linked to aspects of school leadership and management. In this sense, the MSSCI-A not only allows the evaluation of predefined theoretical structures but also enables the exploration of emerging patterns in students’ representations and potentially modifiable elements within educational contexts.

The analyses of internal consistency and convergent validity showed satisfactory results for most dimensions. Alpha and omega coefficients exceeded the recommended thresholds in nearly all factors, indicating high internal homogeneity. Likewise, four factors surpassed the recommended AVE threshold (≥0.50), while six others yielded slightly lower values, although these were still acceptable considering the high reliability and complexity of the model. An important exception was the Organizational Structure factor, which presented low values across all validity and reliability indicators, and therefore, this dimension was excluded from the instrument. This result may reflect students’ lower perceived clarity regarding these institutional aspects, as well as greater heterogeneity in their school experiences. The lack of validity and reliability of this dimension is consistent with the review by Gonzálvez et al. (2023) on school climate instruments, where it was concluded that there is no clear consensus on how to measure this dimension. In this sense, future research should revisit the items within this dimension and explore their relevance in contexts with varying levels of organizational structuring.

Establishing scalar invariance across gender carries significant empirical and practical implications. It provides evidence that men and women interpret both the items and the latent constructs in an equivalent manner, thereby ensuring the validity of latent mean comparisons (Meredith, 1993; Vandenberg & Lance, 2000). This step is essential, as gender-based differences are a recurrent focus of psychological and educational research, and without evidence of invariance such comparisons risk reflecting measurement bias rather than substantive distinctions (Putnick & Bornstein, 2016). In the Latin American context, where persistent gender inequalities influence education, employment, and broader well-being (CEPAL, 2022; OECD, 2021), demonstrating scalar invariance enhances the robustness of empirical findings by confirming that observed differences between men and women are not artifacts of measurement. Consequently, these results strengthen the interpretability of the data and increase their relevance for informing gender-sensitive educational and social policies in the region.

From a contextual perspective, the MSSCI-A addresses a need identified in the Latin American literature: the availability of psychometrically sound and culturally relevant instruments to assess school climate. Unlike many adapted or fragmented scales, this tool was designed from the outset for the local context incorporating dimensions such as pedagogical leadership, student participation, and community interactions, all of which constitute core elements within coexistence and school well-being frameworks promoted in Chile and across the region.

Although the study demonstrated strong psychometric evidence for the MSSCI-A, some limitations should be acknowledged. First, while data were collected across all regions of Chile, participation was not evenly distributed, which suggests that the results should be interpreted with caution in terms of national representativeness. Future studies should aim for more balanced regional participation and replication in other Latin American contexts to evaluate the generalizability of the instrument. Second, although most dimensions showed high levels of reliability and validity, the Organizational Structure factor presented insufficient psychometric properties and was excluded. Further refinement of this dimension and exploration of its conceptual relevance in different educational settings are recommended. Third, the cross-sectional design precludes conclusions about temporal stability; longitudinal studies are necessary to assess measurement invariance across time and the sensitivity of the instrument to change. Finally, while the self-report method allowed for a broad and efficient assessment, potential biases inherent to this approach, such as social desirability, should be considered when interpreting the findings. In addition, future studies could determine the role that psychological variables such as resilience and self-efficacy play in perceptions of the school social climate (Mieres-Chacaltana et al., 2025a, 2025b).

In conclusion, the MSSCI-A emerges as a valid, reliable, and culturally grounded instrument for assessing school social climate among Chilean adolescents. Grounded in an ecological framework, it provides a comprehensive representation of school social climate as a multilevel and relational construct, in line with Bronfenbrenner’s model of human development. The integration of rigorous content validation, advanced statistical techniques, and evidence of scalar invariance across gender positions the scale as a robust contribution to educational measurement. By capturing both theoretically defined and empirically emergent dimensions, the instrument provides a nuanced understanding of how students perceive their school environment. Beyond its psychometric strengths, the MSSCI-A offers practical utility for institutional evaluation, policy monitoring, and the design of interventions aimed at fostering equitable and supportive school climates in Latin America. In addition, its application may contribute to the early identification of factors that could lead to conflict, imbalance, or exclusion within educational settings. Recognizing these aspects in advance allows schools and policymakers to implement preventive measures that strengthen coexistence, psychological well-being, and organizational functioning. From this perspective, the MSSCI-A represents not only a valid and reliable assessment tool but also a practical resource for evidence-based decision-making and continuous improvement in education.

## Figures and Tables

**Table 1:** Multidimensionality of school social climate for students.

Construct	Domain	Dimension	Definition
School Social Climate (SSC)	Safety	Emotional Security	Addresses the existence of psychosocial support and strategies for bullying prevention.
		Discipline	Refers to clarity, fairness, and consistency in the application of rules and conflict resolution.
		Physical Security	Relates to the perception of safety from violence and the presence of preventive measures.
	Community	Collaboration	Participation of family and community members in school life.
		Interpersonal Relationships	Level of trust, support, and bonds among students and school adults.
		Sense of Belonging	A sense of cohesion, belonging, and participation in school activities.
		Respect for Diversity	Recognition of autonomy, equity, and cultural awareness in decision-making.
	Academic	Leadership	Level of support from management teams to teachers and students, including communication and pedagogical guidance.
		Teaching and Learning	Quality of instruction, academic expectations, teaching practices, and student motivation.
	Institutional Environment	Physical Environment	Conditions of the environment, such as lighting, cleanliness, ventilation, and building maintenance.
		Organizational Structure	Factors such as class size, teacher-student ratio, and academic grouping.
		Resources	Access to materials, technology, and equitable distribution of school resources.

Note. Derived from Wang and Degol (2016).

**Table 2:** Preliminary and final items after review by judges based on instrument domains and dimensions.

Domain	Dimension	Preliminary Number of Items	Number of Items After Judges
Safety	Emotional Security	7	10
	Discipline	6	8
	Physical Security	14	14
Community	Collaboration	10	12
	Interpersonal Relationships	8	11
	Sense of Belonging	5	6
	Respect for Diversity	7	7
Academic	Leadership	13	10
	Teaching and Learning	8	8
Institutional Environment	Physical Environment	9	8
	Organizational Structure	6	6
	Resources	16	18
Total		109	118

**Table 3:** Latent correlations among the 11 factors of the MSSCI-A (standardized solution).

	F1	F2	F3	F4	F5	F6	F7	F8	F9	F10	F11
**F1**	1.000	0.666	0.077	0.523	0.568	0.596	0.542	0.571	0.491	0.297	0.269
**F2**		1.000	0.115	0.599	0.666	0.683	0.735	0.705	0.650	0.445	0.372
**F3**			1.000	−0.101	0.203	0.105	0.086	0.175	0.089	0.154	0.19
**F4**				1.000	0.540	0.658	0.664	0.547	0.597	0.336	0.254
**F5**					1.000	0.673	0.678	0.717	0.589	0.425	0.381
**F6**						1.000	0.773	0.696	0.699	0.425	0.357
**F7**							1.000	0.780	0.751	0.518	0.421
**F8**								1.000	0.713	0.460	0.421
**F9**									1.000	0.456	0.457
**F10**										1.000	0.375
**F11**											1.000

Note. All correlations are statistically significant at *p* < 0.05.

**Table 4:** Reliability and Average Variance Extracted.

Factor	α	ω	AVE
F1	0.895	0.895	0.493
F2	0.892	0.889	0.476
F3	0.918	0.916	0.443
F4	0.853	0.838	0.345
F5	0.912	0.905	0.517
F6	0.922	0.920	0.697
F7	0.946	0.945	0.592
F8	0.917	0.917	0.614
F9	0.901	0.897	0.492
F10	0.618	0.621	0.299
F11	0.922	0.918	0.467

Note. α = Cronbach’s alpha; ω = McDonald’s omega; AVE = Average Variance Extracted.

**Table 5:** Measurement invariance by gender.

Model	CFI	TLI	RMSEA	SRMR	∆CFI	∆RMSEA
Configural	0.970	0.969	0.045	0.051	-	-
Metric	0.968	0.968	0.046	0.052	−0.002	0.001
Scalar	0.968	0.967	0.046	0.052	0.000	0.000

Note. CFI = Comparative Fit Index; TLI = Tucker–Lewis Index; RMSEA = Root Mean Square Error of Approximation; SRMR = Standardized Root Mean Square Residual; Δ = difference between nested models.

## Data Availability

The raw data supporting the conclusions of this article will be made available by the authors on request.

## Supplementary Materials

The following supporting information can be downloaded at: https://www.mdpi.com/article/10.3390/bs15111588/s1.

## References

[B1-behavsci-15-01588] Aldridge J., McChesney K. (2018). The relationships between school climate and adolescent mental health and wellbeing: A systematic literature review. International Journal of Educational Research.

[B2-behavsci-15-01588] Bravo-Sanzana M., Bangdiwala S. I., Miranda R. (2021). School violence negative effect on student academic performance: A multilevel analysis. International Journal of Injury Control and Safety Promotion.

[B3-behavsci-15-01588] Bravo-Sanzana M., Miranda-Zapata E., Huaiquián C., Miranda H. (2019a). Clima social escolar en estudiantes de la región de La Araucanía, Chile. Journal of Sport and Health Research.

[B4-behavsci-15-01588] Bravo-Sanzana M., Miranda-Zapata E., Miranda H. (2020a). Psychometric analysis of a school social climate scale: Input elements for the investigation and promotion of well-being. Frontiers in Psychology.

[B5-behavsci-15-01588] Bravo-Sanzana M., Pavez M., Salvo S., Mieres-Chacaltana M. (2019b). Autoeficacia, expectativas y violencia escolar como mediadores del aprendizaje en Matemática. Revista Espacios.

[B6-behavsci-15-01588] Bravo-Sanzana M., Salvo S., Miranda H., Bangdiwala S. (2020b). ¿Qué factores de clima social escolar afectan el desempeño de matemática en estudiantes secundarios? Un análisis multinivel. Culture and Education.

[B7-behavsci-15-01588] Bravo-Sanzana M. V., Varela J., Terán-Mendoza O., Rodríguez-Rivas M. E. (2023). Measuring school social climate in Latin America: The need for multidimensional and multi-informant tests. A systematic review. Frontiers in Psychology.

[B8-behavsci-15-01588] Bronfenbrenner U. (1987). La ecología del desarrollo humano.

[B9-behavsci-15-01588] Bronfenbrenner U. (1989). The developing ecology of human development: Paradigm lost or paradigm regained. Biennial Meeting of the Society for Research in Child Development.

[B10-behavsci-15-01588] Bronfenbrenner U., Vasta R. (1992). Ecological systems theory. Six theories of child development: Revised formulations and current issues.

[B11-behavsci-15-01588] Brown T. A. (2015). Confirmatory factor analysis for applied research.

[B12-behavsci-15-01588] CEPAL (2022). Panorama social de América Latina y el Caribe 2022: La transformación de la educación como base para el desarrollo sostenible. Naciones Unidas–CEPAL.

[B13-behavsci-15-01588] Chen F. F. (2007). Sensitivity of goodness of fit indexes to lack of measurement invariance. Structural Equation Modeling.

[B14-behavsci-15-01588] Chen X., Liao T. (2025). Competitive traits versus (perceived) competitive climates: Disentangling their effects on adolescents’ academic and psychological outcomes. Personality and Individual Differences.

[B15-behavsci-15-01588] Christensen A. P., Golino H. (2021). Estimating the stability of psychological dimensions via bootstrap exploratory graph analysis: A Monte Carlo simulation and tutorial. Psych.

[B16-behavsci-15-01588] Chzhen Y., Leesch J. (2023). Why does school socio-economic composition matter to adolescents’ academic performance? Role of classroom climate and school resources. British Educational Research Journal.

[B17-behavsci-15-01588] Cohen J., McCabe L., Michelli N. M., Pickeral T. (2009). School climate: Research, policy, teacher education and practice. Teachers College Record: The Voice of Scholarship in Education.

[B18-behavsci-15-01588] Del Rey R., Ortega R., Fernández E. (2009). La convivencia escolar: Un modelo de intervención basado en la investigación. Psicothema.

[B19-behavsci-15-01588] Eccles J. S., Roeser R. W. (2011). Schools as developmental contexts during adolescence. Journal of Research on Adolescence.

[B20-behavsci-15-01588] Escobar-Pérez J., Cuervo-Martínez Á. (2008). Validez de contenido y juicio de expertos: Una aproximación a su utilización. Avances en Medición.

[B21-behavsci-15-01588] Ferrando P. J., Lorenzo-Seva U. (2014). El análisis factorial exploratorio de los ítems: Algunas consideraciones adicionales. Anales de Psicología.

[B22-behavsci-15-01588] Franco K., Baumler E., Torres E. D., Lu Y., Wood L., Temple J. R. (2023). The link between school climate and mental health among an ethnically diverse sample of middle school youth. Current Psychology.

[B23-behavsci-15-01588] Golino H., Christensen A. P., Garrido L. E. (2022). Exploratory graph analysis in context. Psicologia: Teoria e Prática.

[B24-behavsci-15-01588] Golino H. F., Epskamp S. (2017). Exploratory graph analysis: A new approach for estimating the number of dimensions in psychological research. PLoS ONE.

[B25-behavsci-15-01588] Gonzálvez C., Bacon V., Kearney C. A. (2023). Systematic and evaluative review of school climate instruments for students, teachers, and parents. Psychology in the Schools.

[B26-behavsci-15-01588] Grazia V., Molinari L. (2021). School climate multidimensionality and measurement: A systematic literature review. Research Papers in Education.

[B27-behavsci-15-01588] Hair J. F., Black W. C., Babin B. J., Anderson R. E. (2019). Multivariate data analysis.

[B28-behavsci-15-01588] Horn J. L. (1965). A rationale and test for the number of factors in factor analysis. Psychometrika.

[B29-behavsci-15-01588] Hu L. T., Bentler P. M. (1999). Cutoff criteria for fit indexes in covariance structure analysis: Conventional criteria versus new alternatives. Structural Equation Modeling.

[B30-behavsci-15-01588] Kalkbrenner M. T. (2021). Alpha, omega, and H internal consistency reliability estimates: Reviewing these options and when to use them. Counseling Outcome Research and Evaluation.

[B31-behavsci-15-01588] Kline R. B. (2016). Principles and practice of structural equation modeling.

[B32-behavsci-15-01588] Lloret-Segura S., Ferreres-Traver A., Hernández-Baeza A., Tomás-Marco I. (2014). El análisis factorial exploratorio de los ítems: Una guía práctica, revisada y actualizada. Anales de Psicología.

[B33-behavsci-15-01588] Lorenzo-Seva U., Timmerman M. E., Kiers H. A. (2011). The Hull Method for selecting the number of common factors. Multivariate Behavioral Research.

[B34-behavsci-15-01588] Lucas-Molina B., Pérez-Albéniz A., Díez-Gómez A., Fonseca-Pedrero E. (2025). Insights into school connectedness: Validation of a scale in Spanish adolescents and relationship with mental health indicators. School Mental Health.

[B35-behavsci-15-01588] Meredith W. (1993). Measurement invariance, factor analysis and factorial invariance. Psychometrika 58.

[B36-behavsci-15-01588] Mieres-Chacaltana M., Salvo-Garrido S., Dominguez-Lara S. (2025a). Modeling the effects of teacher resilience and self-efficacy on prosocialness: Implications for sustainable education. Sustainability.

[B37-behavsci-15-01588] Mieres-Chacaltana M., Salvo-Garrido S., Domínguez-Lara S. (2025b). Resilience and prosociality: Pathways to strengthen teachers’ self-efficacy in the classroom. Frontiers in Psychology.

[B38-behavsci-15-01588] Muñiz J., Fonseca-Pedrero E. (2019). Diez pasos para la construcción de un test. Psicothema.

[B39-behavsci-15-01588] OECD (2021). Latin American Economic Outlook 2021: Working together for a better recovery.

[B40-behavsci-15-01588] Oriol X., Miranda R., Varela J., Garcia N. (2025). Bullying victimization and subjective well-being in 10- and 12-year-old children from 24 countries: The buffering effect of family and teacher support. Child Indicators Research.

[B41-behavsci-15-01588] Podiya J. K., Navaneetham J., Bhola P. (2025). Influences of school climate on emotional health and academic achievement of school-going adolescents in India: A systematic review. BMC Public Health.

[B42-behavsci-15-01588] Posit Team (2025). RStudio: Integrated development environment for R.

[B43-behavsci-15-01588] Preacher K. J., MacCallum R. C. (2003). Repairing Tom Swift’s electric factor analysis machine. Understanding Statistics.

[B44-behavsci-15-01588] Putnick D. L., Bornstein M. H. (2016). Measurement invariance conventions and reporting: The state of the art and future directions for psychological research. Developmental Review.

[B45-behavsci-15-01588] Rudasill K. M., Snyder K. E., Levinson H., Adelson J. L. (2018). Systems view of school climate: A theoretical framework for research. Educational Psychology Review.

[B46-behavsci-15-01588] Thapa A., Cohen J., Guffey S., Higgins-D’Alessandro A. (2013). A review of school climate research. Review of Educational Research.

[B47-behavsci-15-01588] Timmerman M. E., Lorenzo-Seva U. (2011). Dimensionality assessment of ordered polytomous items with parallel analysis. Psychological Methods.

[B48-behavsci-15-01588] Trianes M. V., Blanca M. J., De La Morena L., Infante L., Raya S. (2006). Un cuestionario para evaluar el clima social del centro escolar. Psicothema.

[B49-behavsci-15-01588] Urke H. B., Kristensen S. M., Bøe T., Gaspar de Matos M., Wiium N., Årdal E., Larsen T. (2023). Perceptions of a caring school climate and mental well-being: A one-way street? Results from a random intercept cross-lagged panel model. Applied Developmental Science.

[B50-behavsci-15-01588] Vandenberg R. J., Lance C. E. (2000). A review and synthesis of the measurement invariance literature: Suggestions, practices, and recommendations for organizational research. Organizational Research Methods.

[B51-behavsci-15-01588] Ventura-León J. (2022). De regreso a la validez basada en el contenido. Adicciones.

[B52-behavsci-15-01588] Vieta-Piferrer J., Oriol X., Miranda R. (2024). Correction: Longitudinal associations between cyberbullying victimization and cognitive and affective components of subjective well-being in adolescents: A network analysis. Applied Research Quality Life.

[B53-behavsci-15-01588] Wang M.-T., Degol J. L. (2016). School climate: A review of the construct, measurement, and impact on student outcomes. Educational Psychology Review.

[B54-behavsci-15-01588] Wang M.-T., Eccles J. S. (2012). Adolescent behavioral, emotional, and cognitive engagement trajectories in school and their differential relations to educational success. Journal of Research on Adolescence.

[B55-behavsci-15-01588] Wong M. D., Dosanjh K. K., Jackson N. J., Rünger D., Dudovitz R. N. (2021). The longitudinal relationship of school climate with adolescent social and emotional health. BMC Public Health.

[B56-behavsci-15-01588] World Health Organization (2018). Orientation programme on adolescent health for health-care providers. Handout new modules. In department of child and adolescent health and development.

[B57-behavsci-15-01588] Yamaguchi S., DeVylder J., Yamasaki S., Ando S., Miyashita M., Hosozawa M., Baba K., Niimura J., Nakajima N., Usami S., Kasai K., Hiraiwa-Hasegawa M., Nishida A. (2024). Protective role of school climate for impacts of COVID-19 on depressive symptoms and psychotic experiences among adolescents: A population-based cohort study. Psychological Medicine.

