# Supplementary material for: Design and Validation of the Multidimensional School Social Climate Inventory for Adolescents (MSSCI-A) in Chile
Source: Behav Sci (Basel). 2025 Nov 19;15(11):1588. doi: 10.3390/bs15111588 (PMC12649521; doi:10.3390/bs15111588)
Supplement: Supplementary file 1 [file behavsci-15-01588-s001.zip › Sup Material 2.pdf]

Item	Mean	Median	SD	Skewness	Kurtosis
Puedo expresar mis opiniones sin temor a que alguien me critique o descalifique.	3,456	4	1,155	-0,447	-0,470
Puedo expresar mis sentimientos (como alegría, pena, enojo, miedo), sin que alguien me critique o descalifique.	3,459	4	1,189	-0,454	-0,600
Los/as estudiantes somos respetuosos/as cuando un compañero/a expresa su opinión	3,488	4	1,111	-0,455	-0,374
Puedo dar mi opinión a los/las profesores/as de la escuela sin temor a que me respondan mal o se burlen.	3,782	4	1,138	-0,772	-0,112
Puedo expresar mis emociones a los/las profesoras de la escuela sin temor a que me respondan mal o se burlen.	3,728	4	1,162	-0,712	-0,244
Puedo dar mi opinión al personal directivo de mi escuela (por ejemplo, director/a, inspector/a, orientador/a)sin temor a que me respondan mal o se burlen.	3,681	4	1,154	-0,658	-0,271
Puedo expresar mis emociones al personal directivo de mi escuela (por ejemplo, director/a, inspector/a, orientador/a)sin temor a que me respondan mal o se burlen.	3,630	4	1,160	-0,607	-0,348
Puedo acudir a algún/a compañero/a de mi escuela para pedirle apoyo si tengo algún problema o dificultad.	3,657	4	1,163	-0,690	-0,241
Puedo pedir ayuda a mis profesores/as si tengo algún problema o dificultad.	3,827	4	1,097	-0,848	0,180
Puedo pedir ayuda a las autoridades y directivos de mi escuela (por ejemplo, director/a, inspector/a, orientador/a) si tengo algún problema o dificultad.	3,717	4	1,139	-0,690	-0,183
Tengo claridad de cuáles son las principales reglas y/o normas de mi escuela.	4,097	4	0,954	-1,162	1,318
Tengo claridad de cuáles son las consecuencias de no cumplir las reglas y/o normas en mi escuela.	4,153	4	0,936	-1,223	1,504
Creo que las normas de la escuela son necesarias para convivir	3,953	4	1,005	-0,883	0,458
Creo que es importante que todos los miembros de la comunidad escolar cumplan con las normas de la escuela.	4,078	4	0,955	-1,005	0,828
Los/las estudiantes cumplen las reglas del escuela.	3,212	3	1,082	-0,123	-0,398
Los y las profesores(as) son justos cuando aplican alguna sanción a un/a estudiante en clases.	3,610	4	1,097	-0,523	-0,296
Los y las profesores/as aplican sanciones a los/las estudiantes de manera justa.	3,607	4	1,102	-0,536	-0,283
Las autoridades de la escuela aplican sanciones de manera justa.	3,625	4	1,126	-0,574	-0,275
En mi escuela se realizan actividades para que aprendamos a relacionarnos de formas no violentas (como charlas, obras de teatro, infografías, etc.).	3,855	4	1,084	-0,892	0,296

En mi escuela existen orientaciones sobre cómo resolver los conflictos de maneras no violentas (por ejemplo, mediación entre compañeros/as, conversar del problema).	3,879	4	1,044	-0,876	0,362
Creo que mi escuela es un lugar seguro porque no me siento amenazada/o en riesgo.	3,662	4	1,122	-0,617	-0,230
Acoso o bullying a algún compañero/a dentro de la escuela.	3,736	4	1,194	-0,696	-0,365
Ciberbullying entre compañeros/as (por ejemplo, a través de las redes sociales).	3,757	4	1,259	-0,701	-0,571
Insultos, amenazas o malos tratos verbales entre compañeros/as.	3,256	3	1,328	-0,203	-1,081
Peleas físicas, empujones, golpes entre compañeros/as.	3,346	3	1,229	-0,350	-0,765
Insultos, amenazas o malos tratos verbales de algún/a profesor/a hacia algún/a estudiante.	4,168	5	1,198	-1,339	0,713
Insultos, amenazas o malos tratos verbales de algún/a estudiante hacia algún/a profesor/a.	3,941	4	1,205	-0,972	-0,021
Insultos, amenazas o malos tratos verbales entre adultos en el colegio (personal, autoridades, apoderados/as, etc.).	4,326	5	1,114	-1,643	1,727
Peleas físicas, empujones o golpes de algún/a estudiante hacia algún/a profesor/a.	4,411	5	1,069	-1,876	2,626
Peleas físicas, empujones o golpes de algún/a profesor/a hacia algún/a estudiante.	4,478	5	1,058	-2,054	3,208
Peleas físicas, empujones o golpes entre adultos en el colegio (personal, autoridades, apoderados/as, etc.).	4,493	5	1,043	-2,118	3,515
He sentido temor de venir a la escuela y que me pueda pasar algo (por ejemplo, que me agredan, me asalten o me roben, etc.).	4,187	5	1,210	-1,391	0,817
Mis compañeros/as y yo participamos en las clases (por ejemplo, haciendo preguntas, respondiendo, dando nuestra opinión).	2,636	3	1,019	-0,419	-0,211
He participado en actividades de la escuela como alianzas, celebraciones, eventos deportivos, culturales, bingos, etc.	2,588	3	1,216	-0,453	-0,738
Mis compañeros/as participan en actividades de la escuela (por ejemplo, alianzas, celebraciones, eventos deportivos, culturales, bingos, etc.).	2,857	3	1,005	-0,613	-0,109
He participado en el centro de estudiantes de mi escuela.	1,281	1	1,441	0,679	-0,953
Mis compañeros/as participan en el centro de estudiantes de mi escuela.	2,120	2	1,295	-0,164	-0,946
He participado en la elaboración de normas y/o reglamento de mi escuela.	1,610	2	1,466	0,296	-1,299
Mis compañeros/as participan en la elaboración de normas y/o reglamento de mi escuela.	1,818	2	1,353	0,092	-1,126

Los profesores y profesoras participan en actividades como convivencias, día del alumno, semana del colegio, bingos, actividades culturales, etc.	3,050	3	1,056	-0,970	0,293
Mi apoderado/a asiste a las reuniones de curso y/o entrega de informes.	2,994	3	1,107	-0,897	-0,011
A mi apoderado/a le gusta asistir a las actividades que se realizan en la escuela, como actividades deportivas, culturales, bingos, festivales, etc.	2,261	2	1,289	-0,222	-0,952
Los/las estudiantes nos apoyamos para estudiar.	2,400	2	1,154	-0,284	-0,644
Los/las estudiantes nos apoyamos cuando alguno/a tiene un problema personal.	2,469	3	1,204	-0,383	-0,711
Entre los/las estudiantes.	3,960	5	1,188	-0,583	-0,840
Entre los/las estudiantes y profesores/as.	4,274	5	1,085	-1,077	-0,098
Entre los/las estudiantes y las autoridades o directivos de la escuela (como director/a, inspector/a, orientador/a, etc.).	4,165	5	1,128	-0,871	-0,514
Entre los/las profesores/as.	4,520	5	0,939	-1,688	1,694
Entre los/las profesores/as y las autoridades o directivos de la escuela (como director/a, inspector/a, orientador/a, etc.).	4,424	5	1,002	-1,404	0,747
Entre apoderados/as y profesores/as.	4,317	5	1,039	-1,080	-0,147
Entre apoderados/as y personal de la escuela.	4,325	5	1,040	-1,117	-0,041
Entre las personas que trabajan en la escuela (paradocentes, auxiliares, etc.).	4,437	5	0,988	-1,430	0,868
En general, los distintos miembros de la comunidad escolar (estudiantes, profesores/as, personal, apoderados/as, dirección) tenemos una relación amable entre nosotros.	3,869	4	0,921	-0,740	0,649
En general, los distintos miembros de la comunidad escolar (estudiantes, profesores/as, personal, apoderados/as, dirección) tenemos una relación respetuosa entre nosotros.	3,909	4	0,884	-0,656	0,485
En general, los distintos miembros de la comunidad escolar (estudiantes, profesores/as, personal, apoderados/as, dirección) tenemos una relación de confianza entre nosotros.	3,712	4	0,947	-0,421	-0,011
Me siento valorado/a por profesores/as y autoridades de la escuela.	3,667	4	1,023	-0,574	0,050
La escuela hace sentir que todos/as los/as estudiantes somos importantes.	3,714	4	1,047	-0,590	-0,087
Me siento orgulloso/a de ser estudiante de esta escuela.	3,704	4	1,089	-0,594	-0,163
Siento que pertenezco a esta escuela.	3,696	4	1,092	-0,613	-0,124
Ser parte de esta escuela es importante para mí.	3,631	4	1,125	-0,534	-0,314
Me siento identificado/a con el proyecto educativo de esta escuela.	3,607	4	1,076	-0,480	-0,183

En mi escuela nos enseñan que todas las personas somos importantes.	3,796	4	1,050	-0,674	-0,045
En mi escuela se realizan actividades que nos enseñan a respetar las diferencias entre compañeros/as.	3,768	4	1,049	-0,625	-0,122
En mi escuela todas las personas son tratadas por igual, independiente de las diferencias en el sexo, origen, notas, habilidades etc.	3,842	4	1,074	-0,804	0,118
He sentido que mis compañeros/as me discriminan, por mi sexo, origen, dificultades académicas u otras características.	3,872	5	1,371	-0,847	-0,650
He sentido que mis profesores/as o autoridades me discriminan, por mi sexo, origen, dificultades académicas u otras características.	4,120	5	1,336	-1,205	-0,002
Algunos/as compañeros/as hacen comentarios, chistes o usan insultos que son racistas, sexistas, homofóbicos, etc.**	3,049	3	1,415	-0,030	-1,264
Algunos/as profesores/as, personal o autoridades hacen comentarios, chistes o usan insultos que son racistas, sexistas, homofóbicos, etc.	4,060	5	1,348	-1,131	-0,140
Nos explican qué es lo que se quiere lograr como escuela.	3,593	4	1,159	-0,523	-0,491
Realizan actividades para demostrar lo que se quiere lograr como escuela.	3,600	4	1,126	-0,497	-0,456
Nos comunican cuáles son los valores importantes en la escuela (por ejemplo, el valor de la amistad).	3,748	4	1,095	-0,619	-0,280
Desarrollan diferentes actividades para mostrarnos cuáles son los valores importantes de la escuela (por ejemplo, "no más bullying").	3,780	4	1,100	-0,651	-0,267
Han organizado distintas actividades para que podamos participar (por ejemplo, reuniones con el centro de estudiantes).	3,608	4	1,108	-0,568	-0,216
Nos han pedido nuestra opinión cuando se deben tomar decisiones en la escuela. (por ejemplo, temas relacionados con la convivencia).	3,586	4	1,120	-0,558	-0,282
Están dispuestos a buscar nuevas formas para mejorar la escuela.	3,752	4	1,049	-0,653	0,035
Se preocupan por los problemas que tienen los/las estudiantes.	3,781	4	1,048	-0,673	0,019
Han apoyado a los/as estudiantes que han tenido problemas (por ejemplo, de conducta, económicos, de salud etc.).	3,854	4	1,015	-0,723	0,182
Han apoyado a las familias que han tenido problemas (por ejemplo, económicos, de salud etc.).	3,813	4	1,041	-0,666	0,020
Siento que los/as profesores/as nos tratan con respeto.	4,089	4	0,969	-1,103	1,081

Si nos equivocamos en dar una respuesta en clases los/as profesores/as nos ridiculizan frente a nuestros/as compañeros/as.	3,479	4	1,429	-0,402	-1,182
Siento que puedo dar cualquier opinión en clases porque los/as profesores/as me van a tratar con respeto.	3,882	4	1,015	-0,770	0,265
Cuando doy una respuesta equivocada en una prueba o un trabajo los/as profesores/as me señalan mi error de manera respetuosa.	3,904	4	1,037	-0,859	0,361
Cuando opinamos algo en clases los/as profesores/as nos exigen que seamos respetuosos/as entre nosotros/as.	4,059	4	0,936	-0,960	0,850
Cuando alguien se burla de un/a compañero/a en clases los/as profesores/as le llaman la atención.	3,988	4	1,011	-0,910	0,438
Los/as profesores/as se preocupan de que todos/as podamos participar en clases.	3,934	4	0,984	-0,773	0,303
Cuando hacemos trabajos en grupo los/as profesores/as exigen que respetemos las ideas de todos/as en el grupo.	3,998	4	0,977	-0,855	0,441
El tamaño de las salas de mi escuela es adecuado para que todos/as estemos sentados/as cómodamente.	3,668	4	1,189	-0,666	-0,372
El patio de mi escuela tiene un porte adecuado para que los/as estudiantes puedan correr y jugar.	3,636	4	1,233	-0,645	-0,506
En mi escuela existe un lugar habilitado (casino o sala especial) para que los/as estudiantes podamos comer	3,852	4	1,134	-0,904	0,168
Los espacios de mi escuela están bien iluminados (salas, baños y pasillos).	3,771	4	1,086	-0,692	-0,079
La temperatura de las salas es agradable tanto en invierno como en verano.	3,224	3	1,263	-0,217	-0,891
Los espacios de mi escuela están limpios durante todo el día (baños, salas, patio).	3,354	3	1,196	-0,292	-0,719
Las instalaciones de mi escuela están en buen estado (los baños y juegos funcionan bien, la escuela no se llueve, etc.).	3,322	3	1,219	-0,283	-0,783
Las mesas y sillas de mi sala están en buen estado.	3,340	3	1,201	-0,294	-0,718
La cantidad de estudiantes en la sala de clases permite que todos/as podamos aprender.	3,823	4	1,028	-0,728	0,191
La cantidad de estudiantes en la sala de clases permite que todos/as podamos participar.	3,837	4	0,992	-0,656	0,120
Mi escuela realiza actividades o ceremonias de inicio de clases (por ejemplo, inicio de año).	0,840	1	0,367	-1,850	1,422
Mi escuela realiza actividades conmemorativas (por ejemplo, fiestas patrias, ceremonias religiosas).	0,918	1	0,275	-3,042	7,253
Mi escuela realiza actividades recreativas y/o culturales (por ejemplo, kermeses, obras de teatro, festivales, bingos).	0,816	1	0,387	-1,633	0,667

Mi escuela realiza actividades deportivas (por ejemplo, campeonatos de estudiantes, campeonatos de apoderados/as, competencias interescolares).	0,869	1	0,337	-2,192	2,807
En mi escuela existe un laboratorio de química o biología.	0,616	1	0,486	-0,478	-1,771
Mi escuela tiene una biblioteca.	0,889	1	0,314	-2,480	4,149
La escuela cuenta con asistente social.	0,707	1	0,455	-0,908	-1,176
Mi escuela cuenta con psicólogo/a.	0,770	1	0,421	-1,286	-0,347
Mi escuela cuenta con recursos tecnológicos (como computadores, proyectores y equipos de audio) en buenas condiciones.	3,883	4	1,205	-1,019	0,204
Mi escuela cuenta con recursos tecnológicos (como computadores, proyectores y equipos de audio) suficientes para todos/as los/as estudiantes.	3,737	4	1,239	-0,795	-0,299
El laboratorio de química o biología de mi escuela está bien equipado (tiene microscopios, mecheros, mesas adecuadas).	3,339	4	1,474	-0,461	-1,141
El equipamiento del laboratorio está en buenas condiciones.	3,388	4	1,463	-0,525	-1,064
El equipamiento del laboratorio es suficiente para todos los/as estudiantes cuando trabajan en él.	3,304	4	1,457	-0,438	-1,136
Los libros de lectura obligatoria de la biblioteca están en buenas condiciones.	3,717	4	1,234	-0,822	-0,161
Los libros de lectura obligatoria son suficientes para los/as estudiantes que los necesitan.	3,663	4	1,273	-0,743	-0,411
La biblioteca cuenta con libros o revistas de lectura libre en buenas condiciones.	3,776	4	1,214	-0,903	0,022
En clases de educación física los/as profesores/as y estudiantes cuentan con material deportivo en buenas	3,890	4	1,181	-1,029	0,334
En clases de educación física los/as profesores/as cuentan con material deportivo para todos los estudiantes que lo	3,876	4	1,190	-0,997	0,226
Hay suficientes profesores/as para todos los cursos.	3,924	4	1,176	-1,083	0,452
Los profesores/as siempre asisten a clases.	3,726	4	1,142	-0,750	-0,032
El/la psicólogo/a está disponible para ayudar a los/as estudiantes cuando lo necesitan.	3,757	4	1,307	-0,900	-0,232
El/la asistente social está disponible para ayudar a los/as estudiantes cuando lo necesitan.	3,691	4	1,358	-0,864	-0,397
