# Supplementary material for: Design and Validation of the Multidimensional School Social Climate Inventory for Adolescents (MSSCI-A) in Chile
Source: Behav Sci (Basel). 2025 Nov 19;15(11):1588. doi: 10.3390/bs15111588 (PMC12649521; doi:10.3390/bs15111588)
Supplement: Supplementary file 1 [file behavsci-15-01588-s001.zip › Sup Material 1.pdf]

N° de ítem		Coherence		Clarity		Relevance	
		**Mean**	**Aiken's V**	**Promedio**	**Aiken's V**	**Promedio**	**Aiken's V**
**Emocional security**		**Sufficiency**. Mean= 2.60 Aiken´s V=0.87					
1	Puedo expresar mis opiniones sin temor a ser criticado/a, recibir burlas o malos tratos	3,00	1,00	3,00	1,00	3,00	1,00
2	Puedo expresar mis sentimientos (como alegría, pena, enojo, miedo), sin que alguien se ría de mi, me moleste o me trate mal	3,00	1,00	2,80	0,93	3,00	1,00
3	Los y las estudiantes somos respetuosos/as cuando un compañero/a expresa su opinión	2,60	0,87	2,60	0,87	2,40	0,80
4	Puedo dar mi opinión o expresar mis emociones a los/las profesoras de la escuela sin temor a que me respondan mal o se burlen	3,00	1,00	2,60	0,87	2,60	0,87
5	Confío en que si tengo algún problema o dificultad puedo acudir a alguien de mi escuela para pedir apoyo	3,00	1,00	2,40	0,80	2,40	0,80
6	Confío en que si tengo algún problema, puedo pedir ayuda a mis profesores/as	2,80	0,93	2,80	0,93	2,80	0,93
7	Puedo confiar en las autoridades y directivos de mi escuela (p.e. director, inspector, orientador) si necesito de su ayuda	2,80	0,93	2,40	0,80	2,60	0,87
**Discipline**		**Sufficiency**. Mean= 2.80 Aiken´s V=0.93					
1	**¿Cuán de acuerdo estás con las siguientes afirmaciones?** Tengo claridad de cuáles son las principales reglas de mi escuela, y las consecuencias de no cumplirlas	3,00	1,00	2,60	0,87	3,00	1,00
2	Los y las demás estudiantes de la escuela conocen las reglas y normas de la escuela, y las consecuencias de no cumplirlas	2,80	0,93	2,60	0,87	2,60	0,87
3	Creo que es importante cumplir con las normas de la escuela	3,00	1,00	3,00	1,00	3,00	1,00
4	Los/las estudiantes respetan y cumplen las reglas del escuela	3,00	1,00	2,80	0,93	3,00	1,00
5	**Pensando en el último año, ¿Con qué frecuencia ocurren las siguientes situaciones en tu escuela?** Los y las profesores(as) son justos cuando aplican alguna sanción a un/a estudiante en clases	3,00	1,00	2,40	0,80	3,00	1,00
6	Los y las profesores/as aplican las normas y sanciones a los/las estudiantes de manera justa	2,80	0,93	2,60	0,87	3,00	1,00
**Physical security**		**Sufficiency**. Mean= 3.00 Aiken´s V=1.00					
1	**Pensando en los últimos seis meses, ¿Con qué frecuencia considera usted que ocurren las siguientes situaciones en la escuela?** En mi escuela se realizan actividades para que aprendamos a relacionarnos de formas no violentas (como charlas, obras de teatro, infografías, etc.)	3,00	1,00	2,80	0,93	3,00	1,00
2	**¿Cuán de acuerdo estás con las siguientes afirmaciones?** En mi escuela existen maneras definidas de resolver los conflictos de maneras no violentas (por ejemplo, mediación entre compañeros/as)	2,80	0,93	2,00	0,67	2,80	0,93
3	Situaciones de acoso o bullying a algún compañero/a (en la escuela o por redes sociales)	3,00	1,00	2,60	0,87	3,00	1,00

4	Insultos, amenazas o malos tratos verbales entre compañeros	2,80	0,93	3,00	1,00	3,00	1,00
5	Peleas físicas, empujones, golpes entre compañeros	3,00	1,00	2,80	0,93	3,00	1,00
6	Insultos, amenazas o malos tratos verbales de algún profesor/a a algún estudiante	2,80	0,93	2,80	0,93	2,80	0,93
7	Insultos, amenazas o malos tratos verbales de algún estudiante a algún profesor/a	3,00	1,00	2,80	0,93	3,00	1,00
8	Peleas físicas, empujones, golpes de algún estudiante a algún profesor/a	3,00	1,00	2,80	0,93	3,00	1,00
9	Peleas físicas, empujones, golpes de algún profesor/a a algún estudiante	3,00	1,00	2,80	0,93	3,00	1,00
10	Peleas entre adultos en el colegio (personal, autoridades, apoderados, etc.)	3,00	1,00	2,60	0,87	3,00	1,00
11	**¿Cuán de acuerdo estás con las siguientes afirmaciones?** Creo que la escuela es un lugar seguro, donde no me siento amenazada/o en riesgo	3,00	1,00	2,80	0,93	3,00	1,00
12	Me siento protegido/a cuando estoy al interior de la escuela	2,80	0,93	2,80	0,93	2,80	0,93
13	He sentido miedo de venir a la escuela y me pueda pasar algo (por ejemplo que me agredan, me asalten o me roben, etc.)	2,80	0,93	2,80	0,93	2,80	0,93
14	Hay espacios de mi escuela que son más inseguros (p.e. el patio, los baños)	2,60	0,87	2,20	0,73	2,60	0,87
**Collaboration**		**Sufficiency**. Mean= 3.00 Aiken´s V=1.00					
1	Pensando en el último año, ¿Con qué frecuencia ocurren las siguientes situaciones en tu escuela? Mis compañeros/as y yo participamos activamente en las clases (haciendo preguntas, respondiendo, dando nuestra opinión).	3,00	1,00	3,00	1,00	3,00	1,00
2	Me gusta participar en actividades de la escuela como centro de alumnos, alianzas, celebraciones, eventos deportivos, culturales, bingos, etc.	3,00	1,00	3,00	1,00	3,00	1,00
3	Cuando las escuela hace actividades fuera de clases (alianzas, celebraciones, eventos deportivos, culturales, bingos, etc.), la mayoría de los estudiantes participan.	3,00	1,00	2,80	0,93	3,00	1,00
4	Los profesores y profesoras participan activamente en actividades como convivencias, día del alumno, semana del colegio, bingos, actividades culturales, etc.	3,00	1,00	2,80	0,93	3,00	1,00
5	Mi familia asiste a las reuniones de curso y entrega de informes.	3,00	1,00	2,80	0,93	3,00	1,00
6	¿Cuán de acuerdo te encuentras con las siguientes afirmaciones?A mi familia le gusta asistir a las actividades que se realizan en la escuela, como actividades deportivas, culturales, bingos, festivales, etc.	3,00	1,00	2,80	0,93	3,00	1,00
7	Pensando en el último año, ¿Con qué frecuencia ocurren las siguientes situaciones en tu escuela?Los/las estudiantes nos ayudamos unos con otros, por ejemplo nos apoyamos para estudiar, o cuando algún compañero/a tiene algún problema personal y necesita alguna ayuda.	3,00	1,00	2,60	0,87	3,00	1,00
8	Los/las estudiantes colaboramos con la escuela, ayudando a mantenerla limpia, organizando distintas actividades, proponiendo formas de mejorarla.	3,00	1,00	2,80	0,93	2,80	0,93

9	Las familias colaboran con la escuela, por ejemplo ayudando a mantenerla limpia, reparando espacios, organizando distintas actividades, reuniendo fondos o proponiendo formas de mejorarla.	2,80	0,93	2,40	0,80	2,60	0,87
10	Los distintos miembros de la comunidad escolar (estudiantes, docentes, personal, familias, dirección) colaboramos unos con otros para tener una mejor escuela.	2,80	0,93	2,60	0,87	2,40	0,80
**Interpersonal Relationships**		**Sufficiency**. Mean= 2.80 Aiken´s V=0.93					
1	**¿Cómo considera usted que es la calidad de las relaciones... ?** Entre los/las estudiantes	2,80	0,93	2,40	0,80	3,00	1,00
2	Entre los/las estudiantes y profesores/as	2,80	0,93	2,40	0,80	3,00	1,00
3	Entre los/las estudiantes y las autoridades o directivos de la escuela (como director/a, inspector/a, orientador/a, etc.)	2,80	0,93	2,40	0,80	3,00	1,00
4	Entre los/las profesores/as	2,40	0,80	2,40	0,80	2,40	0,80
5	Entre los/las profesores/as y las autoridades o directivos de la escuela (como director/a, inspector/a, orientador/a, etc.)	2,40	0,80	2,40	0,80	2,40	0,80
6	Entre las familias y profesores/as y personal de la escuela	2,40	0,80	2,40	0,80	2,40	0,80
7	Entre las personas que trabajan en la escuela (profesores/as, paradocentes, auxiliares, etc.)	2,40	0,80	2,40	0,80	2,40	0,80
8	**¿Cuán de acuerdo estás con la siguiente afirmación?** En general, los distintos miembros de la comunidad escolar (estudiantes, profesores, personal, familias, dirección) tenemos una relación amable y respetuosa entre nosotros	3,00	1,00	2,60	0,87	3,00	1,00
**Sense of belonging**		**Sufficiency**. Mean= 2.80 Aiken´s V=0.93					
1	**¿Cuán de acuerdo estás con las siguientes afirmaciones?** Me siento valorado/a por profesores/as y autoridades de la escuela.	3,00	1,00	3,00	1,00	3,00	1,00
2	La escuela hace sentir que todos sus estudiantes son valiosos/importantes.	3,00	1,00	3,00	1,00	3,00	1,00
3	Me siento orgulloso/a de ser estudiante de esta escuela.	3,00	1,00	3,00	1,00	3,00	1,00
4	Siento que pertenezco a esta escuela.	3,00	1,00	3,00	1,00	3,00	1,00
5	Ser parte de esta escuela es importante para mi.	3,00	1,00	3,00	1,00	3,00	1,00
**Respect for Diversity**		**Sufficiency**. Mean= 2.60 Aiken´s V=0.87					
1	**¿Cuán de acuerdo estás con las siguientes afirmaciones?** En mi escuela nos enseñan a apreciar a que todas las personas somos importantes.	3,00	1,00	2,80	0,93	3,00	1,00
2	En mi escuela se realizan actividades que nos enseñan a valorar y respetar las diferencias entre compañeros/as.	3,00	1,00	3,00	1,00	3,00	1,00
3	En mi escuela todas las personas son tratadas por igual, independiente de diferencias de sexo, origen, notas, habilidades etc.	3,00	1,00	3,00	1,00	3,00	1,00
4	**Pensando en el último año, ¿Con qué frecuencia ocurren las siguientes situaciones en tu escuela?** He sentido que mis compañeros/as me tratan mal o discriminan, por mi sexo, origen, dificultades académicas u otras características.	3,00	1,00	2,80	0,93	3,00	1,00

5	He sentido que mis profesores/as o autoridades me tratan mal o discriminan, por por mi sexo, origen, dificultades académicas u otras características.	3,00	1,00	2,80	0,93	3,00	1,00
6	Algunos/as compañeros/as hacen comentarios, chistes o usan insultos que son racistas, sexistas, homofóbicos, etc.	3,00	1,00	3,00	1,00	3,00	1,00
7	Algunos/as profesores/as, personal o autoridades hacen comentarios, chistes o usan insultos que son racistas, sexistas, homofóbicos, etc.	3,00	1,00	3,00	1,00	3,00	1,00
8	Algunos/as estudiantes hacen comentarios, chistes o usan insultos que son racistas, sexistas, homofóbicos, etc.	3,00	1,00	3,00	1,00	3,00	1,00
**Leadership**		**Sufficiency**. Mean= 3.00 Aiken´s V=1.00					
1	**Las autoridades de la escuela (como el director/a, orientador/a o inspector/a):** Nos comunican qué es lo que se quiere lograr como escuela.	2,60	0,87	2,20	0,73	2,40	0,80
2	Realizan actividades para demostrar lo que se quiere lograr como escuela (por ejemplo, la unidad y el respeto en la escuela)	2,60	0,87	1,80	0,60	2,40	0,80
3	Nos dicen cuáles son los valores importantes en la escuela (p.e. el valor de la amistad).	3,00	1,00	2,60	0,87	3,00	1,00
4	Desarrollan diferentes actividades para mostrarnos cuáles son los valores importantes de la escuela (por ejemplo el día del reciclaje, la semana de la alimentación saludable, o no más bullying).	3,00	1,00	2,60	0,87	3,00	1,00
5	**Pensando en el último semestre, ¿con qué frecuencia las autoridades de la escuela (como el director/a, orientador/a o inspector/a)...?** Han organizado distintas instancias formales para que podamos participar por ejemplo, reuniones con el centro de estudiantes, votaciones para elegir talleres que nos gusten, etc.	3,00	1,00	2,80	0,93	3,00	1,00
6	Han organizado distintas instancias formales para que podamos participar en, por ejemplo, la toma de decisiones respecto a la seguridad en la escuela, elegir talleres de convivencia que necesitemos, organizar campañas solidarias.	3,00	1,00	2,80	0,93	3,00	1,00
7	Nos han pedido nuestra opinión cuando se deben tomar decisiones importantes en la escuela.	3,00	1,00	3,00	1,00	3,00	1,00
8	Nos han dado la oportunidades de participar en la toma de decisiones de temas cotidianos de la escuela.	3,00	1,00	2,60	0,87	3,00	1,00
9	Se preocupan por los problemas que tienen los/las estudiantes.	3,00	1,00	2,40	0,80	3,00	1,00
10	Atienden a los/as estudiantes cuando estos lo solicitan.	3,00	1,00	2,40	0,80	3,00	1,00
11	Estarán dispuestos a ayudarme si tengo un problema.	3,00	1,00	2,60	0,87	3,00	1,00
12	Han implementado mecanismos efectivos para apoyar a los estudiantes que han tenido problemas (por ejemplo, de conducta , económicos, de salud etc.).	3,00	1,00	2,60	0,87	3,00	1,00
13	Han implementado mecanismos efectivos para apoyar a las familias que han tenido problemas (por ejemplo, económicos, de salud etc.).	3,00	1,00	2,60	0,87	3,00	1,00
**Teaching and learning**		**Sufficiency**. Mean= 3.00 Aiken´s V=1.00					

1	Siento que los profesores nos tratan con respeto.	3,00	1,00	3,00	1,00	3,00	1,00
2	Si nos equivocamos en dar una respuesta en clases los profesores nos ridiculizan frente a nuestros compañeros. (-)	3,00	1,00	3,00	1,00	3,00	1,00
3	Siento que puedo dar cualquier opinión en clases y los profesores me van a tratar con respeto.	3,00	1,00	3,00	1,00	3,00	1,00
4	Cuando doy una respuesta equivocada en un prueba o un trabajo los profesores me señalan mi error de manera respetuosa.	3,00	1,00	3,00	1,00	3,00	1,00
5	Cuando opinamos algo en clases los profesores nos exigen que seamos respetuoso entre nosotros.	3,00	1,00	3,00	1,00	3,00	1,00
6	Cuando alguien se burla de un compañero en clases los profesores lo reprenden.	2,80	0,93	2,60	0,87	2,60	0,87
7	Los profesores se preocupan de que todos podamos opinar en clases.	3,00	1,00	2,80	0,93	3,00	1,00
8	Cuando hacemos trabajos en grupo los profesores exigen que respetemos las ideas de todos en el grupo.	3,00	1,00	3,00	1,00	3,00	1,00
**Physical Environment**		**Sufficiency**. Mean= 3.00 Aiken´s V=1.00					
1	**¿Cuán de acuerdo está con las siguientes afirmaciones?** Mi escuela, en general, es un lugar cómodo.	3,00	1,00	2,60	0,87	3,00	1,00
2	El tamaño de las salas de mi escuela es adecuado para que todos estemos sentados cómodamente.	3,00	1,00	2,60	0,87	3,00	1,00
3	El patio de mi escuela tiene un porte adecuado para que los/as estudiantes puedan correr y jugar.	3,00	1,00	3,00	1,00	3,00	1,00
4	En mi escuela existe un lugar habilitado (casino o sala especial) para que los/as estudiantes puedan comer cómodamente.	3,00	1,00	2,80	0,93	2,80	0,93
5	Los espacios de mi escuela están bien iluminados (salas, baños y pasillos).	3,00	1,00	3,00	1,00	3,00	1,00
6	La temperatura de las salas es agradable tanto en invierno como en verano.	3,00	1,00	3,00	1,00	3,00	1,00
7	Los espacios de mi escuela están limpios (baños, salas, patio).	3,00	1,00	3,00	1,00	3,00	1,00
8	Las instalaciones de mi escuela están en buen estado (los baños y juegos funcionan bien, la escuela no se llueve).	3,00	1,00	3,00	1,00	3,00	1,00
9	Las mesas y sillas de mi sala están en buen estado.	3,00	1,00	3,00	1,00	3,00	1,00
**Organizational Structure**		**Sufficiency**. Mean= 3.00 Aiken´s V=1.00					
1	**¿Cuán de acuerdo está con las siguientes afirmaciones?** La cantidad de estudiantes en la sala de clases permite que todos/as puedan aprender.	3,00	1,00	3,00	1,00	3,00	1,00
2	La cantidad de estudiantes en la sala de clases permite que todos/as puedan participar.	3,00	1,00	3,00	1,00	3,00	1,00
3	Indique si en su escuela se realizan o no las siguientes actividades.Mi escuela realiza actividades o ceremonias de inicio clases (por ejemplo, inicio de año).	3,00	1,00	2,80	0,93	3,00	1,00
4	Mi escuela realiza actividades conmemorativas (por ejemplo, fiestas patrias, ceremonias religiosas).	3,00	1,00	3,00	1,00	3,00	1,00

5	Mi escuela realiza actividades recreativas y/o culturales (por ejemplo, kermeses, obras de teatro, festivales, bingos).	3,00	1,00	3,00	1,00	3,00	1,00
6	Mi escuela realiza actividades deportivas (por ejemplo, campeonatos de estudiantes, campeonatos de apoderados, competencias interescolares).	3,00	1,00	3,00	1,00	3,00	1,00
**Resources**		**Sufficiency**. Mean= 2.80 Aiken´s V=0.93					
1	**¿Cuán de acuerdo está con las siguientes afirmaciones?** Mi escuela cuenta con recursos tecnológicos (como computadores, proyectores y equipos de audio) en buenas condiciones.	3,00	1,00	3,00	1,00	3,00	1,00
2	Mi escuela cuenta con recursos tecnológicos (como computadores, proyectores y equipos de audio) suficientes para todos los estudiantes.	3,00	1,00	3,00	1,00	3,00	1,00
3	En mi escuela existe un laboratorio.	3,00	1,00	2,80	0,93	2,40	0,80
4	El laboratorio de mi escuela está equipado (tiene microscopios, mecheros, mesas adecuadas).	3,00	1,00	3,00	1,00	2,40	0,80
5	El equipamiento del laboratorio está en buenas condiciones.	3,00	1,00	3,00	1,00	2,40	0,80
6	El equipamiento del laboratorio es suficiente para todos los estudiantes cuando trabajan en él.	3,00	1,00	3,00	1,00	2,40	0,80
7	Mi escuela tiene una biblioteca.	3,00	1,00	3,00	1,00	3,00	1,00
8	Los libros de lectura obligatoria de la biblioteca están en buenas condiciones.	3,00	1,00	3,00	1,00	3,00	1,00
9	Los libros de lectura obligatoria son suficientes para los estudiantes que los necesitan.	3,00	1,00	3,00	1,00	3,00	1,00
10	La biblioteca cuenta con libros o revistas de lectura libre están en buenas condiciones.	3,00	1,00	3,00	1,00	3,00	1,00
11	En clases de educación física los/as profesores/as y estudiantes cuentan con material deportivo en buenas condiciones.	3,00	1,00	3,00	1,00	3,00	1,00
12	En clases de educación física los/as profesores/as cuentan con material deportivo para todos los estudiantes que lo requieren.	3,00	1,00	3,00	1,00	3,00	1,00
13	Hay suficientes profesores/as para todos los cursos.	3,00	1,00	3,00	1,00	3,00	1,00
14	Los profesores/as siempre asisten a clases.	3,00	1,00	3,00	1,00	3,00	1,00
15	Mi escuela cuenta con psicólogo/a.	2,80	0,93	2,80	0,93	3,00	1,00
16	El/la psicólogo/a está disponible para ayudar a los/as estudiantes cuando lo necesitan.	3,00	1,00	3,00	1,00	3,00	1,00
