# Supplementary material for: Design and Validation of the Multidimensional School Social Climate Inventory for Adolescents (MSSCI-A) in Chile
Source: Behav Sci (Basel). 2025 Nov 19;15(11):1588. doi: 10.3390/bs15111588 (PMC12649521; doi:10.3390/bs15111588)
Supplement: Supplementary file 1 [file behavsci-15-01588-s001.zip › Sup Material 3.pdf]

Item	F1	F2	F3	F4	F5	F6	F7	F8	F9	F10	F11
Puedo expresar mis opiniones sin temor a que alguien me critique o descalifique.	**0,338**	0,009	-0,002	0,043	0,012	0,000	0,000	0,000	0,000	0,000	0,000
Puedo expresar mis sentimientos (como alegría, pena, enojo, miedo), sin que alguien me critique o descalifique.	**0,409**	0,021	0,000	0,008	0,005	0,019	0,000	0,000	0,000	0,000	0,000
Los/as estudiantes somos respetuosos/as cuando un compañero/a expresa su opinión **	0,168	0,103	0,026	0,050	0,055	0,000	0,000	0,000	0,008	0,000	0,000
Puedo dar mi opinión a los/las profesores/as de la escuela sin temor a que me respondan mal o se burlen.	**0,453**	0,012	0,005	0,003	0,000	0,002	0,000	0,030	0,000	0,000	0,000
Puedo expresar mis emociones a los/las profesoras de la escuela sin temor a que me respondan mal o se burlen.	**0,474**	0,028	0,000	0,002	0,006	0,022	0,000	0,028	0,000	0,000	0,000
Puedo dar mi opinión al personal directivo de mi escuela (por ejemplo, director/a, inspector/a, orientador/a)sin temor a que me respondan mal o se burlen.	**0,495**	0,037	0,000	0,000	0,015	0,005	0,000	0,000	0,000	0,000	0,000
Puedo expresar mis emociones al personal directivo de mi escuela (por ejemplo, director/a, inspector/a, orientador/a)sin temor a que me respondan mal o se burlen.	**0,551**	0,011	0,000	0,001	0,000	0,000	0,000	0,000	0,000	0,000	0,000
Puedo acudir a algún/a compañero/a de mi escuela para pedirle apoyo si tengo algún problema o dificultad.	**0,316**	0,016	0,000	0,081	0,011	0,014	0,000	0,000	0,000	0,000	0,000
Puedo pedir ayuda a mis profesores/as si tengo algún problema o dificultad.	**0,427**	0,044	0,000	0,025	0,005	0,035	0,000	0,022	0,000	0,000	0,000
Puedo pedir ayuda a las autoridades y directivos de mi escuela (por ejemplo, director/a, inspector/a, orientador/a) si tengo algún problema o dificultad.	**0,391**	0,102	0,000	0,000	0,030	0,016	0,012	0,000	0,002	0,000	0,001
Tengo claridad de cuáles son las principales reglas y/o normas de mi escuela.	0,005	**0,419**	0,000	0,026	0,018	0,007	0,008	0,023	0,001	0,000	0,005
Tengo claridad de cuáles son las consecuencias de no cumplir las reglas y/o normas en mi escuela.	0,015	**0,391**	0,009	0,039	0,000	0,000	0,000	0,029	0,004	0,000	0,000
Creo que las normas de la escuela son necesarias para convivir.	0,044	**0,409**	0,000	0,013	0,005	0,020	0,013	0,018	0,000	0,000	0,001
Creo que es importante que todos los miembros de la comunidad escolar cumplan con las normas de la escuela.	0,024	**0,428**	0,000	0,041	0,023	0,000	0,000	0,018	0,000	0,000	0,000
Los/las estudiantes cumplen las reglas del escuela.**	0,084	0,161	0,001	0,076	0,032	0,001	0,000	0,000	0,043	0,000	0,000

Los y las profesores(as) son justos cuando aplican alguna sanción a un/a estudiante en clases.	0,021	**0,463**	0,000	0,002	0,015	0,021	0,003	0,004	0,031	0,000	0,000
Los y las profesores/as aplican sanciones a los/las estudiantes de manera justa.	0,008	**0,531**	0,000	0,000	0,001	0,009	0,004	0,013	0,003	0,000	0,000
Las autoridades de la escuela aplican sanciones de manera justa.	0,046	**0,436**	0,000	0,000	0,006	0,007	0,026	0,000	0,028	0,006	0,008
En mi escuela se realizan actividades para que aprendamos a relacionarnos de formas no violentas (como charlas, obras de teatro, infografías, etc.).	0,006	**0,344**	0,000	0,013	0,000	0,000	0,120	0,004	0,011	0,033	0,005
En mi escuela existen orientaciones sobre cómo resolver los conflictos de maneras no violentas (por ejemplo, mediación entre compañeros/as, conversar del problema).	0,047	**0,379**	0,000	0,024	0,000	0,002	0,071	0,006	0,006	0,000	0,015
Creo que mi escuela es un lugar seguro porque no me siento amenazada/o en riesgo.	0,077	0,218	0,031	0,014	0,025	0,062	0,029	0,000	0,057	0,000	-0,002
Acoso o bullying a algún compañero/a dentro de la escuela.	0,000	0,006	**0,430**	0,000	0,000	0,000	0,000	0,000	0,000	0,000	0,000
Ciberbullying entre compañeros/as (por ejemplo, a través de las redes sociales).	0,000	0,004	**0,435**	0,000	0,000	0,000	0,000	0,000	0,000	0,000	0,000
Insultos, amenazas o malos tratos verbales entre compañeros/as.	0,008	0,025	**0,487**	0,012	0,012	0,000	0,000	0,000	0,007	0,000	0,000
Peleas físicas, empujones, golpes entre compañeros/as.	0,000	0,011	**0,410**	0,000	0,006	0,000	0,000	0,000	0,000	0,000	0,000
Insultos, amenazas o malos tratos verbales de algún/a profesor/a hacia algún/a estudiante.	0,000	0,000	**0,435**	0,000	0,000	0,003	0,007	0,015	0,000	0,000	0,000
Insultos, amenazas o malos tratos verbales de algún/a estudiante hacia algún/a profesor/a.	0,000	0,000	**0,461**	0,000	0,009	0,000	0,002	0,000	0,007	0,000	0,000
Insultos, amenazas o malos tratos verbales entre adultos en el colegio (personal, autoridades, apoderados/as, etc.).	0,000	0,000	**0,477**	0,000	0,003	0,000	0,000	0,006	0,000	0,000	0,008
Peleas físicas, empujones o golpes de algún/a estudiante hacia algún/a profesor/a.	0,000	0,000	**0,474**	-0,005	0,001	0,000	0,000	0,000	0,000	0,000	0,000
Peleas físicas, empujones o golpes de algún/a profesor/a hacia algún/a estudiante.	0,000	-0,006	**0,497**	-0,022	0,005	0,000	0,000	0,003	0,000	0,015	0,004
Peleas físicas, empujones o golpes entre adultos en el colegio (personal, autoridades, apoderados/as, etc.).	0,000	-0,017	**0,501**	-0,013	0,010	0,000	0,000	0,000	0,000	0,005	0,000

He sentido temor de venir a la escuela y que me pueda pasar algo (por ejemplo, que me agredan, me asalten o me roben, etc.).	0,000	0,024	**0,319**	0,000	0,014	0,000	0,000	0,000	0,000	0,000	0,000
Mis compañeros/as y yo participamos en las clases (por ejemplo, haciendo preguntas, respondiendo, dando nuestra opinión).	0,046	0,036	0,000	**0,336**	0,036	0,012	0,006	0,014	0,007	0,000	0,000
He participado en actividades de la escuela como alianzas, celebraciones, eventos deportivos, culturales, bingos, etc.	0,023	0,000	0,000	**0,405**	0,004	0,027	0,002	0,000	0,000	0,000	0,000
Mis compañeros/as participan en actividades de la escuela (por ejemplo, alianzas, celebraciones, eventos deportivos, culturales, bingos, etc.).	0,000	0,013	0,000	**0,422**	0,026	0,000	0,011	0,018	0,016	0,005	0,000
He participado en el centro de estudiantes de mi escuela.	0,000	0,018	-0,083	**0,325**	-0,004	0,000	0,003	0,000	0,008	0,000	0,000
Mis compañeros/as participan en el centro de estudiantes de mi escuela.	0,000	0,005	0,000	**0,345**	0,003	0,000	0,032	0,000	0,004	0,000	0,000
He participado en la elaboración de normas y/o reglamento de mi escuela.	0,000	0,005	-0,055	**0,465**	0,000	0,011	0,020	0,000	0,013	0,000	0,000
Mis compañeros/as participan en la elaboración de normas y/o reglamento de mi escuela.	0,000	0,010	-0,020	**0,482**	0,000	0,004	0,041	0,000	0,016	0,000	0,000
Los profesores y profesoras participan en actividades como convivencias, día del alumno, semana del colegio, bingos, actividades culturales, etc.	0,019	0,072	0,043	0,234	0,042	0,000	0,031	0,048	0,010	0,014	0,003
Mi apoderado/a asiste a las reuniones de curso y/o entrega de informes.	0,000	0,033	0,018	0,254	0,040	0,000	0,003	0,000	0,000	0,000	0,000
A mi apoderado/a le gusta asistir a las actividades que se realizan en la escuela, como actividades deportivas, culturales, bingos, festivales, etc.	0,007	0,007	-0,006	**0,370**	0,004	0,036	0,005	0,000	0,004	0,000	0,000
Los/las estudiantes nos apoyamos para estudiar.	0,032	0,046	0,001	**0,406**	0,042	0,055	0,014	0,000	0,017	0,000	0,000
Los/las estudiantes nos apoyamos cuando alguno/a tiene un problema personal.	0,080	0,001	0,000	**0,366**	0,049	0,022	0,035	0,000	0,001	0,000	0,000
Entre los/las estudiantes.**	0,052	0,037	0,030	0,089	0,188	0,016	0,000	0,000	0,015	0,004	0,000
Entre los/las estudiantes y profesores/as.	0,007	0,002	0,002	0,001	**0,393**	0,020	0,010	0,022	0,003	0,003	0,010
Entre los/las estudiantes y las autoridades o directivos de la escuela (como director/a, inspector/a, orientador/a, etc.).	0,027	0,007	0,006	0,000	**0,402**	0,034	0,022	0,000	0,004	0,000	0,001

Entre los/las profesores/as.	0,000	0,009	0,024	0,014	**0,419**	0,000	0,000	0,032	0,000	0,008	0,004
Entre los/las profesores/as y las autoridades o directivos de la escuela (como director/a, inspector/a, orientador/a, etc.).	0,003	0,011	0,009	0,015	**0,499**	0,000	0,001	0,007	0,000	0,006	0,004
Entre apoderados/as y profesores/as.	0,000	0,000	0,000	0,006	**0,435**	0,024	0,001	0,011	0,002	0,000	0,003
Entre apoderados/as y personal de la escuela.	0,007	0,000	0,000	0,004	**0,507**	0,009	0,007	0,007	0,004	0,002	0,003
Entre las personas que trabajan en la escuela (paradocentes, auxiliares, etc.).	0,000	0,002	0,001	0,030	**0,443**	0,008	0,000	0,000	0,000	0,000	0,011
En general, los distintos miembros de la comunidad escolar (estudiantes, profesores/as, personal, apoderados/as, dirección) tenemos una relación amable entre nosotros.	0,018	0,015	0,000	0,028	**0,416**	0,046	0,036	0,028	0,023	0,000	0,003
En general, los distintos miembros de la comunidad escolar (estudiantes, profesores/as, personal, apoderados/as, dirección) tenemos una relación respetuosa entre nosotros.	0,004	0,012	0,000	0,036	**0,382**	0,025	0,019	0,045	0,010	0,000	0,003
En general, los distintos miembros de la comunidad escolar (estudiantes, profesores/as, personal, apoderados/as, dirección) tenemos una relación de confianza entre nosotros.**	0,024	0,034	0,000	0,024	0,289	0,083	0,053	0,001	0,002	0,000	0,000
Me siento valorado/a por profesores/as y autoridades de la escuela.**	0,056	0,013	0,000	0,033	0,117	0,281	0,029	0,063	0,003	0,000	0,000
La escuela hace sentir que todos/as los/as estudiantes somos importantes.	0,017	0,022	0,003	0,010	0,050	**0,322**	0,130	0,034	0,023	0,004	-0,001
Me siento orgulloso/a de ser estudiante de esta escuela.	0,001	0,026	0,000	0,008	0,020	**0,503**	0,049	0,015	0,024	0,023	0,004
Siento que pertenezco a esta escuela.	0,028	0,015	0,000	0,026	0,016	**0,509**	0,015	0,015	0,004	0,000	0,000
Ser parte de esta escuela es importante para mí.	0,002	0,000	0,000	0,029	0,000	**0,538**	0,026	0,000	0,015	0,000	0,000
Me siento identificado/a con el proyecto educativo de esta escuela.	0,004	0,048	0,000	0,054	0,045	**0,426**	0,049	0,000	0,020	0,000	0,004
En mi escuela nos enseñan que todas las personas somos importantes.	0,000	0,024	0,000	0,009	0,008	0,112	**0,344**	0,039	0,019	0,006	0,000
En mi escuela se realizan actividades que nos enseñan a respetar las diferencias entre compañeros/as.	0,000	0,058	0,000	0,007	0,016	0,039	**0,390**	0,015	0,003	0,024	0,002

En mi escuela todas las personas son tratadas por igual, independiente de las diferencias en el sexo, origen, notas, habilidades etc.**	0,000	0,028	0,009	0,032	0,036	0,018	0,195	0,062	0,032	0,000	0,002
He sentido que mis compañeros/as me discriminan, por mi sexo, origen, dificultades académicas u otras características.	0,000	0,000	**0,441**	-0,003	0,000	0,000	0,000	0,000	0,000	0,000	0,000
He sentido que mis profesores/as o autoridades me discriminan, por mi sexo, origen, dificultades académicas u otras características.	0,000	-0,008	**0,507**	-0,025	0,016	0,000	0,000	0,016	-0,031	0,000	0,011
Algunos/as compañeros/as hacen comentarios, chistes o usan insultos que son racistas, sexistas, homofóbicos, etc.**	0,021	0,009	0,263	0,001	0,000	0,000	0,000	0,000	0,000	0,000	0,000
Algunos/as profesores/as, personal o autoridades hacen comentarios, chistes o usan insultos que son racistas, sexistas, homofóbicos, etc.	-0,002	-0,002	**0,466**	-0,020	0,001	0,000	0,000	0,010	-0,004	0,014	0,015
Nos explican qué es lo que se quiere lograr como escuela.	0,000	0,013	0,000	0,012	0,008	0,020	**0,403**	0,009	0,009	0,020	0,009
Realizan actividades para demostrar lo que se quiere lograr como escuela.	0,000	0,009	0,000	0,028	0,000	0,008	**0,503**	0,000	0,019	0,017	0,000
Nos comunican cuáles son los valores importantes en la escuela (por ejemplo, el valor de la amistad).	0,000	0,019	0,000	0,008	0,005	0,031	**0,467**	0,026	0,010	0,009	0,009
Desarrollan diferentes actividades para mostrarnos cuáles son los valores importantes de la escuela (por ejemplo, “no más bullying”).	0,000	0,024	0,000	0,000	0,000	0,000	**0,462**	0,015	0,013	0,016	0,001
Han organizado distintas actividades para que podamos participar (por ejemplo, reuniones con el centro de estudiantes).	0,000	0,036	0,000	0,036	0,020	0,001	**0,351**	0,000	0,026	0,032	0,008
Nos han pedido nuestra opinión cuando se deben tomar decisiones en la escuela. (por ejemplo, temas relacionados con la convivencia).	0,000	0,010	0,000	0,038	0,018	0,013	**0,401**	0,000	0,032	0,010	0,006
Están dispuestos a buscar nuevas formas para mejorar la escuela.	0,000	0,007	0,000	0,000	0,005	0,036	**0,440**	0,028	0,031	0,003	0,009
Se preocupan por los problemas que tienen los/las estudiantes.	0,002	0,043	0,000	0,008	0,014	0,037	**0,390**	0,055	0,035	0,002	0,010
Han apoyado a los/as estudiantes que han tenido problemas (por ejemplo, de conducta, económicos, de salud etc.).	0,010	0,008	0,000	0,014	0,010	0,005	**0,432**	0,051	0,016	0,001	0,007
Han apoyado a las familias que han tenido problemas (por ejemplo, económicos, de salud etc.).	0,000	0,005	0,000	0,020	0,013	0,006	**0,356**	0,032	0,006	0,002	0,005
Siento que los/as profesores/as nos tratan con respeto.	0,000	0,044	0,028	0,009	0,060	0,039	0,067	**0,311**	0,057	0,000	0,008

Si nos equivocamos en dar una respuesta en clases los/as profesores/as nos ridiculizan frente a nuestros/as compañeros/as.	0,006	0,000	0,174	-0,035	0,000	0,000	0,000	0,000	-0,016	0,000	0,001
Siento que puedo dar cualquier opinión en clases porque los/as profesores/as me van a tratar con respeto.	0,058	0,002	0,000	0,014	0,012	0,054	0,007	**0,381**	0,042	0,006	0,001
Cuando doy una respuesta equivocada en una prueba o un trabajo los/as profesores/as me señalan mi error de manera respetuosa.	0,005	0,008	0,000	0,004	0,012	0,002	0,018	**0,424**	0,036	0,000	0,001
Cuando opinamos algo en clases los/as profesores/as nos exigen que seamos respetuosos/as entre nosotros/as.	0,000	0,027	0,007	0,019	0,034	0,000	0,028	**0,449**	0,024	0,008	0,003
Cuando alguien se burla de un/a compañero/a en clases los/as profesores/as le llaman la atención.	0,008	0,009	0,009	0,017	0,008	0,000	0,042	**0,437**	0,033	0,000	0,016
Los/as profesores/as se preocupan de que todos/as podamos participar en clases.	0,005	0,010	0,000	0,002	0,011	0,030	0,074	**0,415**	0,050	0,000	0,007
Cuando hacemos trabajos en grupo los/as profesores/as exigen que respetemos las ideas de todos/as en el grupo.	0,000	0,009	0,000	0,012	0,008	0,004	0,072	**0,462**	0,055	0,000	0,009
El tamaño de las salas de mi escuela es adecuado para que todos/as estemos sentados/as cómodamente.	0,000	0,008	0,000	0,000	0,000	0,003	0,025	0,025	**0,436**	0,000	0,014
El patio de mi escuela tiene un porte adecuado para que los/as estudiantes puedan correr y jugar.	0,000	0,018	0,000	0,000	0,000	0,020	0,040	0,023	**0,368**	0,010	0,021
En mi escuela existe un lugar habilitado (casino o sala especial) para que los/as estudiantes podamos comer cómodamente.	0,002	0,009	0,000	0,010	0,004	0,000	0,043	0,085	0,282	0,000	0,035
Los espacios de mi escuela están bien iluminados (salas, baños y pasillos).	0,000	0,003	0,000	0,005	0,000	0,001	0,033	0,043	**0,461**	0,004	0,036
La temperatura de las salas es agradable tanto en invierno como en verano.	0,000	0,029	-0,021	0,031	0,000	0,017	0,013	0,000	**0,360**	0,000	0,013
Los espacios de mi escuela están limpios durante todo el día (baños, salas, patio).	0,006	0,037	-0,008	0,004	0,000	0,000	0,005	0,015	**0,399**	0,005	0,001
Las instalaciones de mi escuela están en buen estado (los baños y juegos funcionan bien, la escuela no se llueve, etc.).	0,000	0,018	-0,006	0,005	0,000	0,008	0,016	0,000	**0,507**	0,000	0,017
Las mesas y sillas de mi sala están en buen estado.	0,001	0,039	-0,004	0,012	0,000	0,011	0,008	0,000	**0,393**	0,000	0,021
La cantidad de estudiantes en la sala de clases permite que todos/as podamos aprender.	0,001	0,005	0,007	0,008	0,036	0,005	0,033	0,056	**0,417**	0,005	0,004

La cantidad de estudiantes en la sala de clases permite que todos/as podamos participar.	0,001	0,020	0,000	0,021	0,022	0,030	0,027	0,066	**0,387**	0,000	0,012
Mi escuela realiza actividades o ceremonias de inicio de clases (por ejemplo, inicio de año).	0,000	0,001	0,000	0,000	0,008	0,006	0,043	0,000	0,005	**0,329**	0,024
Mi escuela realiza actividades conmemorativas (por ejemplo, fiestas patrias, ceremonias religiosas).	0,000	0,000	0,022	0,010	0,008	0,000	0,000	0,010	0,000	**0,345**	0,013
Mi escuela realiza actividades recreativas y/o culturales (por ejemplo, kermeses, obras de teatro, festivales, bingos).	0,000	0,022	0,000	0,004	0,000	0,013	0,029	0,000	0,010	**0,332**	0,040
Mi escuela realiza actividades deportivas (por ejemplo, campeonatos de estudiantes, campeonatos de apoderados/as, competencias interescolares).	0,000	0,004	0,000	0,000	0,000	0,001	0,026	0,000	0,002	**0,449**	0,031
En mi escuela existe un laboratorio de química o biología. **	0,000	-0,002	-0,002	0,000	0,000	-0,001	0,000	0,000	0,000	0,000	0,192
Mi escuela tiene una biblioteca.**	0,000	0,000	0,000	0,000	0,000	0,000	0,000	0,000	0,003	0,036	0,114
La escuela cuenta con asistente social.**	0,000	0,000	0,000	0,000	0,000	0,000	0,000	0,000	0,000	0,028	0,276
Mi escuela cuenta con psicólogo/a.**	0,000	0,000	0,000	0,000	0,000	0,000	0,000	0,000	0,005	0,020	0,264
Mi escuela cuenta con recursos tecnológicos (como computadores, proyectores y equipos de audio) en buenas condiciones.	0,000	0,003	0,007	0,000	0,006	0,003	0,007	0,003	0,023	0,018	**0,409**
Mi escuela cuenta con recursos tecnológicos (como computadores, proyectores y equipos de audio) suficientes para todos/as los/as estudiantes.	0,000	0,000	0,000	0,000	0,000	0,000	0,000	0,000	0,035	0,005	**0,447**
El laboratorio de química o biología de mi escuela está bien equipado (tiene microscopios, mecheros, mesas adecuadas).	0,000	0,000	0,000	0,000	0,000	0,000	0,000	0,000	0,000	0,000	**0,477**
El equipamiento del laboratorio está en buenas condiciones.	0,000	0,000	0,000	0,000	0,000	0,000	0,000	0,000	0,000	0,000	**0,535**
El equipamiento del laboratorio es suficiente para todos los/as estudiantes cuando trabajan en él.	0,000	0,000	0,000	0,000	0,000	0,000	0,000	0,000	0,010	0,000	**0,466**
Los libros de lectura obligatoria de la biblioteca están en buenas condiciones.	0,000	0,001	0,000	0,000	0,000	0,000	0,007	0,000	0,013	0,000	**0,479**
Los libros de lectura obligatoria son suficientes para los/as estudiantes que los necesitan.	0,000	0,001	0,000	0,000	0,000	0,005	0,008	0,000	0,021	0,008	**0,422**

La biblioteca cuenta con libros o revistas de lectura libre en buenas condiciones.	0,000	0,000	0,000	0,000	0,002	0,000	0,000	0,010	0,007	0,022	**0,514**
En clases de educación física los/as profesores/as y estudiantes cuentan con material deportivo en buenas condiciones.	0,000	0,000	0,008	0,000	0,007	0,000	0,000	0,000	0,016	0,013	**0,460**
En clases de educación física los/as profesores/as cuentan con material deportivo para todos los estudiantes que lo requieran.	0,000	0,002	0,004	0,000	0,000	0,000	0,000	0,011	0,021	0,004	**0,484**
Hay suficientes profesores/as para todos los cursos.	0,000	0,000	0,023	0,003	0,011	0,000	0,002	0,018	0,009	0,000	**0,436**
Los profesores/as siempre asisten a clases.**	0,000	0,000	0,000	0,000	0,014	0,000	0,000	0,009	0,024	0,000	0,297
El/la psicólogo/a está disponible para ayudar a los/as estudiantes cuando lo necesitan.	0,001	0,013	0,000	0,000	0,004	0,000	0,024	0,000	0,000	0,009	**0,448**
El/la asistente social está disponible para ayudar a los/as estudiantes cuando lo necesitan.	0,000	0,017	0,000	0,000	0,001	0,000	0,023	0,000	0,000	0,000	**0,460**

Note. **Item dropped
